# Association between the *AKT1* single nucleotide polymorphism (rs2498786, rs2494752 and rs5811155) and microscopic polyangiitis risk in a Chinese population

**DOI:** 10.1007/s00438-023-02012-6

**Published:** 2023-04-07

**Authors:** Lizhen Li, Jinlan Rao, Jingjing Lan, Yan Zhu, Aimei Gong, Liepeng Chu, Fei Feng, Chao Xue

**Affiliations:** 1 Department of Nephrology, the Second Affiliated Hospital of Guangxi Medical University, Nanning, Guangxi China; 2 Department of Nephrology, Laboratory of Kidney Disease of Hunan Provincial People’s Hospital, the First-Affiliated Hospital of Hunan Normal University, Changsha, 410005 People’s Republic of China; 3 Department of Nephrology, the First Hospital of Foshan City, Foshan, China; 4 Department of Nephrology, Hengyang Medical School, the First Affiliated Hospital, University of South China, Hengyang, Hunan China; 5 Department of Nephrology, the First Affiliated Hospital of Guangxi University of Traditional Chinese Medicine, Nanning, Guangxi China

**Keywords:** AKT1, ANCA-associated vasculitis, Microscopic polyangiitis (MPA), Gene polymorphism, Single Nucleotide Polymorphisms (SNPs)

## Abstract

Microscopic polyangiitis (MPA) is an autoimmune disease, characterized by ANCA in blood and necrotizing inflammation of small and medium-sized vessels, one of the three clinical phenotypes of ANCA-associated vasculitis (AAV). Autophagy has been confirmed to be involved in the pathogenesis of AAV. AKT1 is one of the autophagy-regulated proteins. Its single nucleotide polymorphisms (SNPs) are associated with multiple immune-related diseases, but there are rarely studies in AAV. The incidence rate of AAV has a notable geographic difference, and MPA is predominant in China. The aim of this study was to investigate the association between *AKT1* SNP and MPA risk. Genotypes of 8 loci in *AKT1* were evaluated by multiplex polymerase chain reaction (PCR) and high-throughput sequencing in 416 people, including 208 MPA patients and 208 healthy volunteers from Guangxi in China. Additionally, data of 387 healthy volunteers from China were obtained from the 1000Genomes Project on public database. Differences were observed between the loci (rs2498786, rs2494752, and rs5811155) genotypes in *AKT1* and MPA risk (*P* = 7.0 × 10^–4^, *P* = 3.0 × 10^–4^, and *P* = 5.9 × 10^–5^, respectively). A negative association was detected in the Dominant model (*P* = 1.2 × 10^–3^, *P* = 2.0 × 10^–4^ and *P* = 3.6 × 10^–5^, respectively). A haplotype (G-G-T) was associated with MPA risk negatively (*P* = 7.0 × 10^–4^). This study suggests that alleles (rs2498786 G, rs2494752 G and rs5811155 insT) are protective factors for MPA and alleles (rs2494752 G and rs5811155 insT) for MPO-ANCA in patients with MPA. There is a haplotype (G-G-T), which is a protective factor for MPA. It suggests that the role of AKT1 in MPA/AAV needs further study to provide more intervention targets for MPA/AAV.

**Supplementary Information:**

The online version contains supplementary material available at 10.1007/s00438-023-02012-6.

## Introduction

ANCA-associated vasculitis (AAV) is a systemic autoimmune disease by serum antineutrophil cytoplasmic antibodies (ANCA), inflammatory injury to endothelial cells and vascular tissue and leading to lung and kidney damage (Jennette et al. 2013). AAV includes microscopic polyangiitis (MPA), granulomatosis with polyangiitis (GPA) and eosinophilic granulomatosis with polyangiitis (EGPA). The prevalence rate of AAV is 300–421 per million persons (Berti et al. 2017). A survey reported that the AAV incidence rate was 0.25‰ among inpatients in China in 2015 (Li et al. 2018). Although the incidence rate of AAV is very low, the mortality rate without treatment is up to 80% within one year (Li et al. 2016b). Although hormones combined with immunosuppression has greatly improved the prognosis of AAV, secondary infection caused by this regimen has become the main cause of death (Li et al. 2016b). Therefore, it is necessary to provide precision medicine treatment by discovering the pathogenesis of AAV.

The pathogenesis of AAV is complex and not fully understood. ANCA was confirmed to play important role in the process (Nakazawa et al. 2019). And the type of ANCA is related to the clinical subtype. MPO/pANCA is predominant in the MPA patients (Kronbichler et al. 2020). Genetic factors also were found to be associated with AAV including MPA (Trivioli et al. 2022). There are four genome-wide association studies (GWAS) to detecte the associaton between SNPs and AAV (Kawasaki and Tsuchiya 2021). Among these GWAS, HLA-DQB1 SNP (rs1049072) was found to be associated with MPA risk and MPO/pANCA strongly (Merkel et al. 2017). Although not correlated with MPA, HLA-DQB1 (rs5000634) is significantly correlated with MPO-ANCA (Lyons et al. 2012). Gene polymorphism of some candidate genes may be associated with MPA risk such as CTLA4 (rs3087243) (Kamesh et al. 2009; Rahmattulla et al. 2016), IL10 (rs1800896) (Bártfai et al. 2003), TLR2 (rs3804100) (Wu et al. 2015), FCGR3B (copy number variation) (Fanciulli et al. 2007), KIR2DS3 (carrier) (Miyashita et al. 2006), LILRA2 (rs2241524) (Mamegano et al. 2008), TERT (rs2736100) and DSP (rs2076295) (Kawasaki et al. 2020). When it comes to MPO-ANCA, TERT (rs2736100), DSP (rs2076295) (Kawasaki et al. 2020), DQA1*0302, DQB1*0303, DQB1*0201, DQA1*0501(Wang et al. 2019), CTLA4(rs231775) (Slot et al. 2008), IRF5 (rs10954213) (Kawasaki et al. 2013), TLR2 (rs3804099 and rs3804100) (Guo et al. 2015), STAT4(rs7574865) (Rahmattulla et al. 2016), ITGB2(rs235326) (Gencik et al. 2000) and MPO (rs2333227) (Reynolds et al. 2002) were candidate genes.

Autophagy is a biological process in the eukaryotic cells to maintain cell homeostasis. It is related to immunity (Jang et al. 2019) and diseases (Mizushima and Levine 2020) in human, also with AAV (Tang et al. 2015). Autophagy gene polymorphisms are confirmed to be associated with multiple immune-related diseases. RAC (Rho family)-alpha serine/threonine-protein kinase (AKT1), encoded by the gene *AKT1* (position:14q32.33) (Staal et al. 1988), is involved in the regulation of autophagy. Autophagy was regulated by phosphorylated AKT1 by inhibiting AKT1S1 and RPTOR to activate mTORC1 (Saxton and Sabatini 2017) and by stabling the Drosophila melanogaster protein Acinus (Acn) (Nandi et al. 2014). There are increasing studies on the AKT1 gene polymorphism involving multiple diseases related to immune disorder (Pereira et al. 2014; Cheng et al. 2016; Li et al. 2016a). However, little is reported regarding the association between *AKT1* SNP and the AAV or MPA risk.

Studies reports that MPA is more common than GPA and eGPA in Asian population (Berti et al. 2017; Geetha and Jefferson 2020), and ANCA status was found to be related to clinical phenotypes (Lyons et al. 2019). Previous studies on autophagopathies suggest that autophagy gene polymorphisms can provide precision medicine for human disease (Grosjean et al. 2022). This study was designed to investigate the association between *AKT1* SNPs and MPA risk in a Chinese population to provide precision medicine for this disease. MPO-ANCA status in MPA patients were also involved in the stratified analysis.

## Patients and methods

### Study design and participants

416 participants were recruited at the Second Affiliated Hospital of Guangxi Medical University between September 2009 and April 2020. It included 208 adult MPA patients and 208 healthy adult volunteers. The criteria for recruitment were as follows (a) MPA was diagnosed, based on the 2012 Chapel Hill Consensus Conference of Vasculitis (Jennette et al. 2013), and (b) absence of other diagnosis, such as, malignancy, infection, and drugs. In addition, 387 healthy adult volunteers were recruited from Chinese 1000genomes online project database (International Genome Sample Resource). The demographic and clinical characteristics of all participants are listed in Table 1.

**Table 1:** Characteristics of participants

Characteristic	MPA group (*n* = 208)	Control group (*n* = 595)	
		Guangxi (*n* = 208)	1000Genomes (*n* = 387)
Gender (M/F)	78/130	81/127	184/203
Age (years, mean ± SD)	54.87 ± 14.75	51.15 ± 12.67	–
BVAS (mean ± SD)	16.84 ± 4.47	–	–
MPO-ANCA ( +)	141 (67.7%)	–	–
PR3-ANCA( +)	16 (7.7%)	–	–
Renal biopsy	89 (42.7%)	–	–

The MPA group consisted of 208 MPA patients recruited from the Second Affiliated Hospital of Guangxi Medical University. The Control group consisted of two parts. Data from the Guangxi patient population collected at the Second Affiliated Hospital of Guangxi Medical University, and 1000Genomes obtained from an online database named the International Genome Sample Resource (https://www.internationalgenome.org)

*BVAS* Birmingham Vasculitis Activity Score

*AKT1* SNPs were collected from the 1000 Genomes Project and the gnomAD Genome Project. 8 loci (Supplement Table 1) in *AKT1* were selected to detect genotypes, as their allele frequencies differ in Asian, European, and African populations. This difference is similar to the different incidence rate of MPA in these populations.

### Data extraction

The blood samples (416) collection was as follows: for MPA patients, samples were collected at the time of MPA diagnosis. For healthy volunteers, sample collection was during physical examinations at the Second Affiliated Hospital of Guangxi Medical University. Genomic DNA was isolated from peripheral blood samples using the Blood DNA Extraction Kit (Tiangen, Beijing, CA), following manufacturer’s protocols, and quantified via Nanodrop 2000 spectrophotometer (Thermo Fisher Scientific, Waltham, MA, USA).

### High throughput sequencing and genotype analysis

A primer pool containing four loci was designed and synthesized in our laboratory. The target gene was amplified via PCR. Following amplification, the products were detected via agarose gel electrophoresis, and purified using AMPure XP beads. Next, the products were recovered for a second PCR analysis, with labeled primers. The second PCR products were then purified and recovered using AMPure XP beads, as mentioned before. Sequencing was performed using HiSeq XTen (Sangon Biotech, Shanghai, CA), then processed by cutadapt (V 1.2.1), and finally detected by PRINSEQ-lite (v 0.20.3). The clean DNA-seq reads were aligned to the AKT1 genome using BWA (v 0.7.13-r1126), then genotypes of loci in AKT1 were computed using samtools (v 0.1.18).

### Statistical analysis

Data analysis was conducted via Chi-square or Fisher’s exact test, and the relative risk was computed using logistic regression analysis via SNPStats (web tool for SNP analysis). Linkage disequilibrium and Haplotype analysis were performed via Haploview (version 4.1). A total of 8 *AKT1* SNPs chosen from the 1000Genomes Project were tested in this study. The* P* value threshold for this study is1.56 × 10^–3^ (0.05/32) with Bonferroni correction.

## Results

### Participant characteristics

This study recruited 208 MPA patients and 595 healthy adult volunteers. No discernible difference was observed in gender distribution between the MPA and Control groups. The BVAS (mean ± SD) of all MPA patients are presented in Table 1. Overall, 141 out of 208 patients exhibited serum MPO-ANCA ( +) and PR3-ANCA ( +), and there were 89 MPA patients who underwent kidney biopsy.

### Associations between the AKT1 genotypes and MPA risk

Genotypes of 8 *AKT1* loci in 416 participants from Guangxi and 387 Chinese in 1000genomes Project were collected. Three of them (rs2498786, rs2494752 and rs5811155) were associated with MPA risk. The information of the three loci is presented in Table 2. The lowest minor allele frequency (MAF) was 0.22 in the MPA group, and 0.15 in the Control group. All allele frequencies conformed to the MAF requirement (MAF > 0.05). In this study, the Hardy–Weinberg equilibrium (HWE) varied between 0.08 and 0.58, and conformed to the HWE requirement (HWE > 0.05) Table 3.

**Table 2:** Information of AKT1 alleles (MPA group, *n* = 208, Control group, *n* = 595)

Loci	Variant type	Group	Ref	Alt(%)	MAF	HWE (*p*-value)
rs2498786	SNV	MPA group	355 (85%)	61 (15%)	0.15	0.58
	C > G	Control group	917 (77%)	273 (23%)	0.23	0.56
rs2494752	SNV	MPA group	325 (78%)	91 (22%)	0.22	0.42
	A > G	Control group	802 (67%)	388 (33%)	0.33	0.08
rs5811155	Insertion	MPA group	–	94 (23%)	0.23	0.56
	insT	Control group	–	417 (35%)	0.35	0.11

The HEW was calculated in the control group and MPA group, respectively, using the Pearson’s chi-squared test in SNPStats (web tool for SNP analysis)

*SNV* Single Nucleotide Variation. *Ref* Ref Allele. *Alt* Alt Allele. *MAF* Minor Allele Frequency. *HWE* Hardy–Weinberg equilibrium

**Table 3:** Association between the AKT1 genotypes and MPA risk (*n* = 803, adjusted by sex)

Loci	Model	Genotype	Control group (*n* = 595)	MPA group (*n* = 208)	OR (95% CI)	*P*-value
rs2498786	Codominant	CC	356 (59.8%)	150 (72.1%)	1.00	–
		C/G	205 (34.5%)	55 (26.4%)	0.63 (0.44–0.90)	**7.0 × 10**^–4^*
		G/G	34 (5.7%)	3 (1.4%)	0.21 (0.06–0.69)	
	Dominant	CC	356 (59.8%)	150 (72.1%)	1.00	–
		C/G-G/G	239 (40.2%)	58 (27.9%)	0.57 (0.41–0.81)	**1.2 × 10**^–3^*
	Recessive	C/C–C/G	561 (94.3%)	205 (98.6%)	1.00	–
		G/G	34 (5.7%)	3 (1.4%)	0.24 (0.07–0.79)	4.8 × 10^–3^
	Overdominant	C/C-G/G	390 (65.5%)	153 (73.6%)	1.00	–
		C/G	205 (34.5%)	55 (26.4%)	0.68 (0.48–0.97)	2.9 × 10^–2^
rs2494752	Codominant	A/A	280 (47.1%)	129 (62%)	1.00	–
		A/G	242 (40.7%)	67 (32.2%)	0.60 (0.43–0.85)	**3.0 × 10**^–4^**
		G/G	73 (12.3%)	12 (5.8%)	0.36 (0.19–0.68)	
	Dominant	A/A	280 (47.1%)	129 (62.0%)	1.00	–
		A/G-G/G	315 (52.9%)	79 (38.0%)	0.55 (0.40–0.76)	**2.0 × 10**^–4^**
	Recessive	A/A-A/G	522 (87.7%)	196(94.2%)	1.00	–
		G/G	73 (12.3%)	12 (5.8%)	0.44 (0.23–0.83)	5.8 × 10^–3^
	Overdominant	A/A-G/G	353 (59.3%)	141 (67.8%)	1.00	–
		A/G	242 (40.7%)	67 (32.2%)	0.70 (0.50–0.97)	3.2 × 10^–2^
rs5811155	Codominant	C/C	260 (43.7%)	126 (60.6%)	1.00	–
		C/T	253 (42.5%)	70 (33.6%)	0.57 (0.41–0.80)	**5.9 × 10**^–5^**
		T/T	82 (13.8%)	12 (5.8%)	0.31 (0.16–0.58)	
	Dominant	C/C	260 (43.7%)	126 (60.6%)	1.00	–
		C/T-T/T	335 (56.3%)	82 (39.4%)	0.51 (0.37–0.70)	**3.6 × 10**^–5^**
	Recessive	C/C- C/T	513 (86.2%)	196 (94.2%)	1.00	–
		T/T	82 (13.8%)	12 (5.8%)	0.39 (0.21–0.73)	**1.1 × 10**^–3^*
	Overdominant	C/C-T/T	342 (57.5%)	138 (66.3%)	1.00	–
		C/T	253 (42.5%)	70 (33.6%)	0.68 (0.49–0.95)	2.4 × 10^–2^

Data analysis was performed via SNPStats (web tool for SNP analysis). The* P* value threshold is 1.56 × 10^–3^ (0.05/32) with Bonferroni correction. *P-value is less than 1.56 × 10^–3^ (0.05/32). **P-value is less than 3.13 × 10^–4^ (0.01/32)

Four models (Codominant, Dominant, Recessive and Overdominant models) were calculated to detect the association between *AKT1* SNPs and the MPA risk. Negative association were observed between the MPA risk and rs2498786 G in the Codominant model (*P* = 7.0 × 10^–4^) and Dominant model (*P* = 1.2 × 10^–3^). Significant negative association was observed between the MPA risk and the alleles (rs2494752 G and rs5811155insT) in the Codominant model (*P* = 3.0 × 10^–4^ and *P* = 5.9 × 10^–5^) and Dominant model (*P* = 2.0 × 10^–4^ and *P* = 3.6 × 10^–5^). The negative association also was found in alleles (rs5811155 insT) in the Recessive model (*P* = 1.1 × 10^–3^).

Myeloperoxidase-ANCA (MPO-ANCA) were positive in 141 out of 208 MPA patients. Associations between *AKT1* SNPs and MPO-ANCA were performed in stratified analysis (Table 4). Negative association was observed between the MPO-ANCA and rs2494752 G in the Codominant model (*P* = 5.0 × 10^–4^) and significant negative association in the Dominant model (*P* = 2.0 × 10^–4^). Significant negative association were observed between the MPO-ANCA and rs5811155 insT in the Codominant and Dominant models (*P* = 1.0 × 10^–4^). While no association was observed between the MPO-ANCA and rs2498786.

**Table 4:** Association between the AKT1 genotypes and MPO-ANCA + in MPA patients (*n* = 736, adjusted by sex)

Loci	Model	Genotype	Control group (*n* = 595)	MPO-ANCA + in MPA group (*n* = 141)	OR (95% CI)	*P*-value
rs2498786	Codominant	CC	356 (59.8%)	103 (73.0%)	1.00	–
		C/G	205 (34.5%)	36 (25.5%)	0.60 (0.39–0.90)	2.3 × 10^–3^
		G/G	34 (5.7%)	2 (1.4%)	0.20 (0.05–0.85)	
	Dominant	CC	356 (59.8%)	103 (73.0%)	1.00	–
		C/G-G/G	239 (40.2%)	38 (26.9%)	0.54 (0.36–0.81)	2.3 × 10^–3^
	Recessive	C/C–C/G	561 (94.3%)	139 (98.6%)	1.00	–
		G/G	34 (5.7%)	2 (1.4%)	0.24 (0.06–0.99)	1.5 × 10^–2^
	Overdominant	C/C-G/G	390 (65.5%)	105 (74.5%)	1.00	–
		C/G	205 (34.5%)	36 (25.5%)	0.64 (0.42–0.97)	3.2 × 10^–2^
rs2494752	Codominant	A/A	280 (47.1%)	91 (64.5%)	1.00	–
		A/G	242 (40.7%)	42 (29.8%)	0.53 (0.36–0.80)	**5.0 × 10**^–4^*
		G/G	73 (12.3%)	8 (5.7%)	0.34 (0.16–0.73)	
	Dominant	A/A	280 (47.1%)	91 (64.5%)	1.00	–
		A/G-G/G	315 (52.9%)	50 (35.5%)	0.49 (0.33–0.72)	**2.0 × 10**^–4^**
	Recessive	A/A-A/G	522 (87.7%)	133 (94.3%)	1.00	
		G/G	73 (12.3%)	8 (5.7%)	0.43 (0.20–0.91)	1.6 × 10^–2^
	Overdominant	A/A-G/G	353 (59.3%)	99 (70.2%)	1.00	–
		A/G	242 (40.7%)	42 (29.8%)	0.62 (0.42–0.92)	1.6 × 10^–2^
rs5811155	Codominant	C/C	260 (43.7%)	88 (62.4%)	1.00	–
		C/T	253 (42.5%)	45 (31.9%)	0.52 (0.35–0.78)	**1.0 × 10**^–4^**
		T/T	82 (13.8%)	8 (5.7%)	0.29 (0.13–0.62)	
	Dominant	C/C	260 (43.7%)	88 (62.4%)	1.00	–
		C/T-T/T	335 (56.3%)	53 (37.6%)	0.47 (0.32–0.68)	**1.0 × 10**^–4^**
	Recessive	C/C- C/T	513 (86.2%)	133 (94.3%)	1.00	–
		T/T	82 (13.8%)	8 (5.7%)	0.38 (0.18–0.80)	4.8 × 10^–3^
	Overdominant	C/C-T/T	342 (57.5%)	96 (68.1%)	1.00	–
		C/T	253 (42.5%)	45 (31.9%)	0.63 (0.43–0.93)	1.8 × 10^–2^

Data analysis was performed via SNPStats (web tool for SNP analysis). The* P* value threshold is 1.56 × 10^–3^ (0.05/32) with Bonferroni correction. *P-value is less than 1.56 × 10^–3^ (0.05/32). **P-value is less than 3.13 × 10^–4^ (0.01/32)

### AKT1 SNP data

**Fig. 1:**
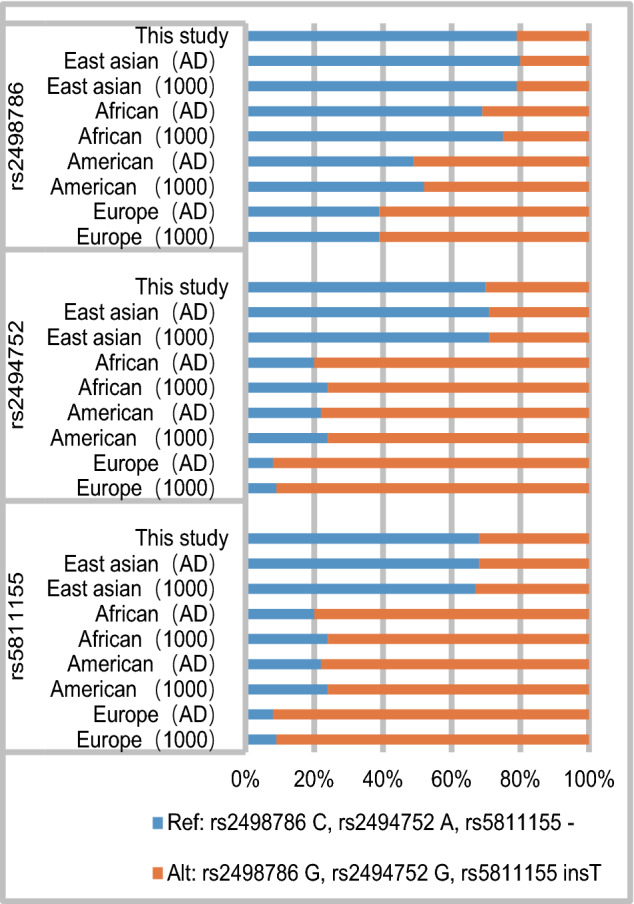
Distribution of AKT1 alleles (rs2498786, rs2494752 and rs5811155). The figure illustrates the allelic distribution among various races within the 1000Genomes, gnomAD-Genomes, and this study databases (East asian, African, American, and Europe). The 1000Genomes and gnomAD-Genomes data were obtained from public database (National Center for Biotechnology Information and International Genome Sample Resource). *AD gnomAD-Genomes* 1000, 1000Genomes

The allele frequencies of the loci (rs2498786, rs2494752 and rs5811155) in this study are shown in Fig. 1. The allele frequencies in different races (East Asian, African, American, and Europe) in the 1000Genomes and gnomAD-Genomes Project are also shown in Fig. 1. The allele frequencies in this study were similar to those in East Asian in the two projects, and it was significantly different from those in Europe, African or American.

The three loci (rs2498786, rs2494752, and rs5811155) locate in the upstream region of the *AKT1* gene (Supplement Table 1). Summaries of the functionality scores for these loci were obtained from the 3DSNP website (omic.tech) (Supplement Fig. 1). Rs2498786 is located in the promoter region of *AKT1*, and as candidate cis-regulatory elements (cCREs) in 54 distinct cell types. Rs2494752 is located in the enhancer state in 60 distinct cell types and rs5811155 in 72. Integrated regulation in the three loci from ENCODE is presented in Supplement Fig. 2. In cell lines derived from blood or vascular tissue, four histone modifications (CTCF, H3K4Me1, H3K4Me3, and H3K27Ac) and DNase sensitivity related to the three loci and gene regulation are shown in Figs. A and C. The three loci are involved in different histone modifications and have different DNase I sensitivity in the same cell line. Information about transcription factor binding related to the three loci is shown in Fig. B. The transcription factors binding to the regions where the three loci are located are partially the same.

Expression quantitative trait locus (eQTL) analysis of these loci were obtained from the 3DSNP website (omic.tech) (Supplement Fig. 3). Genotype of rs2498786 can influence AKT1 expression in whole blood and SIVA1 in whole blood, artery aorta and artery tibial tissue. Genotype of rs2494752 can influence AKT1 expression in whole blood tissue and SIVA1 in artery aorta tissue. And genotype of rs5811155 can influence AKT1 expression in whole blood tissue and SIVA1 in artery aorta tissue.

### Linkage disequilibrium and Haplotype analysis

**Fig. 2:**
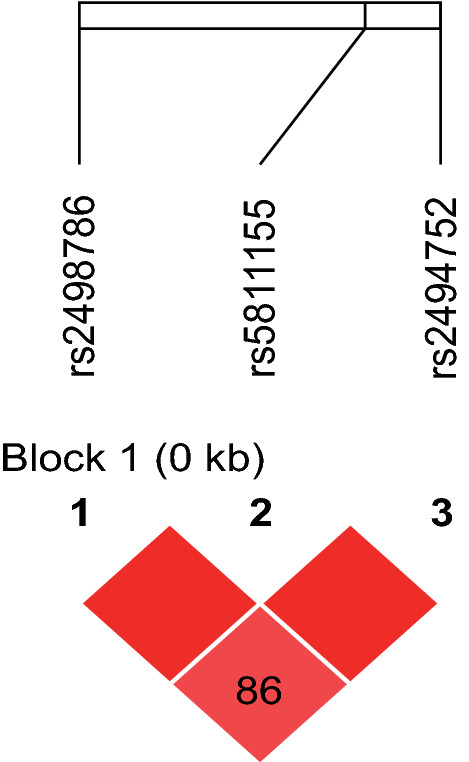
The D' and R^2^ value heat maps of linkage disequilibrium in rs2498786, rs2494752, and rs5811155*.* D’ (rs2498786, rs5811155) = 1.0; D' (rs2498786, rs2494752) = 0.86; D’ (rs5811155, rs2494752) = 1.0. R^2^ (rs2498786, rs5811155) = 0.56; R^2^ (rs2498786, rs2494752) = 0.46; R^2^ (rs5811155, rs2494752) = 0.91. Data analysis was performed via Haploview (version 4.1)

Linkage disequilibrium and allelic haplotypes were calculated in this study. The D' and R2 value heat maps of the linkage disequilibrium involving rs2498786, rs2494752, and rs5811155 are presented in Fig. 2. The linkage disequilibrium existed in all three loci in this study (D'(rs2498786, rs5811155) = 1.0; D'(rs2498786, rs2494752) = 0.86; D'(rs5811155, rs2494752) = 1.0. R2 (rs2498786, rs5811155) = 0.56; R2 (rs2498786, rs2494752) = 0.46; R2 (rs5811155, rs2494752) = 0.91).

Haplotypes (G-G-T and C-G-T) were negatively associated with the MPA risk (*P* = 7.0 × 10^–4^ and *P* = 8.3 × 10^–3^), but G-A-T was positive (Table 5). Considering the* P* value threshold (1.56 × 10^–3^) in this study, people with the haplotype G-G-T is a protective factor for MPA.

**Table 5:** Haplotype of alleles (rs2498786, rs2494752, and rs5811155) (*n* = 803)

Haplotype	Freq (%)	MPA (%)	Control (%)	OR (95% CI)	*P*-value
C-A –	68.2	77.4	65.0	1.00	–
G-G-T	18.8	14.0	20.5	0.58 (0.42—0.79)	**7.0 × 10**^–4^*
C-G-T	11.0	8.0	12.1	0.59 (0.40—0.87)	8.3 × 10^–3^
G-A-T	2.0	7.2	2.4	0.25 (0.08—0.82)	2.3 × 10^–2^

Data analysis was performed via SNPStats (web tool for SNP analysis). Global haplotype association* p*-value: 1.0** × 10**^**–4**^. The *P* value threshold is 1.56 × 10^–3^ (0.05/32) with Bonferroni correction. * P value is less than 1.56 × 10^–3^ (0.05/32)

*Freq* Frequency. *MPA* MPA group. Control, Control group

## Discussion

In this study, association between *AKT1* SNPs and MPA risk / MPO-ANCA were calculated. Results show that alleles (rs2498786 G, rs2494752 G and rs5811155 insT) are protective factors for MPA and alleles (rs2494752 G and rs5811155 insT) for MPO-ANCA in MPA patients. Moreover, there is a haplotype (G-G-T) which is a protective factor for MPA.

Allele frequencies of rs2498786 G, rs2494752 G and rs5811155 insT are obviously lower in East Asian populations than European in the 1000Genomes and gnomAD-Genomes Project. This is in contrast to the high incidence rate of MPA in Chinese or Japanese populations belonging to East Asia, and low incidence of MPA in Europeans (Berti et al. 2017; Geetha and Jefferson 2020). In this study, the allele frequencies are very close to those of East Asians in the 1000Genomes and gnomAD-Genomes Project. And negative association are found between rs2498786 G, rs2494752 G, rs5811155 insT and the MPA risk. These suggest that alleles (rs2498786 G, rs2494752 G and rs5811155 insT) may be protective factors for MPA in Chinese population.

The three loci (rs2498786, rs2494752 and rs5811155) locate in the upstream region of *AKT1* (NCBI). Annotation shows that rs2498786 locates in the promoter area of *AKT1*, and both rs2494752 and rs5811155 locate in enhancer area. Liu S.Y. et al. reported that the AKT1 protein levels of the genotype GG of rs2498786 is significantly elevated, compared to the GC and CC in a study involving type 2 diabetes mellitus with Alzheimer’s Disease (Liu et al. 2015). Both Li X. et al. and Wang M.Y. et al. reported that rs2494752 G significantly increases *AKT1* promoter activity to influence the expression of AKT1 protein (Wang et al. 2016; Li et al. 2017). These suggest that genotypes of these loci may influence the expression of AKT1 protein. eQTL analysis shows that the three loci influence the expression of AKT1 in whole blood cells including neutrophils, dendritic cells and macrophages et.

Neutrophil is the initial and key cell in the formation of NETs and ANCAs which has been demonstrated in the common pathways of the pathogenesis of AAV including MPA (Nakazawa et al. 2019). ANCAs are produced due to the intolerance to NETs activated by neutrophils (Thiam et al. 2020b). MPO and PR3 receptors upregulate on the plasma membrane in primed neutrophils, which can bind to ANCAs to promote NETs formation (Kronbichler et al. 2020). These lead to more production of ANCAs and NETs. In vitro culture of neutrophils, NETs can be induced by reactive oxygen species (ROS) (Thiam et al. 2020a), but they cannot be produced without ROS (Kenny et al. 2017). These suggest that ROS released by neutrophil is essential for the production of NETs and ANCA.

ROS was released by the NADPH oxidase complex in neutrophils (El-Benna et al. 2016). In this process, PI3K was activated by Gβγ dissociated from the membrane (Houslay et al. 2016) and phosphorylated Src homologous domains (Volmering et al. 2016). In the PI3K/AKT1/mTOR signaling pathway, AKT1 phosphorylated by PI3K activates mTORC1 to regulate autophagy levels (Saxton and Sabatini 2017). Sha L.L et al. found that autophagy was induced by ANCAs and promote NETs formation in AAV (Sha et al. 2016). In addition, Chen J et al. reported that *AKT1*-deficient neutrophils exhibit a small, but significant, increase in ROS production (Chen et al. 2010). Liu G et al. reported that *AKT1* knockout mice exhibit markedly increased ROS in model of acute inflammatory lung injury (ALI) and Staphylococcus aureus infection (Liu et al. 2013). These suggest that AKT1 may be involved in the formation of NETs and ANCAs by regulating neutrophil-mediated ROS.

The antigens necessary for the formation of NET and ANCA are presented by dendritic cells (DCs) to CD4 + T cells (Sangaletti et al. 2012). Na ( +)/H ( +) exchangers in DCs require AKT1 to be activated by LPS or oxidative stress (Zhou et al. 2015) to maintain their PH and function. And AKT1 is also required for pro-inflammatory signal-mediated DCs survival and maturation (Park et al. 2006). In addition, Vegting et al. reported that M2-type macrophages are increased in vitro and vivo studies related to AAV (Vegting et al. 2021). And Duan et al. reported that M2 associated gene of macrophage was regulated by the PI3K/ AKT1/mTOR signaling pathway (Duan et al. 2020). These suggest that AKT1 may be involved in the pathogenesis of AAV by regulating functions of DCs and macrophages in blood.

There is a linkage imbalance inheritance at the loci (rs2498786, rs2494752 and rs5811155) in this study. The haplotype (G-G-T) is found to be a protective factor for MPA. It is consistent with the results that rs2498786 G, rs2494752 G and rs5811155 insT are protective factors for MPA. However, a haplotype (G-A-T) is observed to be a risk factor for MPA in this study. Results showed that rs2498786 G and rs5811155 insT are protective factors, but rs2494752 A is not. ANCA status is related to genotype of rs2494752 and strongly to rs5811155, but not to rs2498786. Integrated regulation from ENCODE (UCSC Browser) shows that in the same cell line, the three loci are involved in different histone modifications and have different DNase sensitivity. And only one transcription factor can bind to the region where all three sites are located. These indicate that the signal of association may derive from one of these SNPs rather than the Linkage disequilibrium inheritance of the loci.

Besides, eQTL analysis shows that loci (rs2498786, rs2494752 and rs5811155) can also influence the expression of SIVA1 in vascular tissues. It suggests that SNPs in the three loci (rs2498786, rs2494752 and rs5811155) may affect AAV by influencing the SIVA1 expression in vascular tissues, which is the target tissue for major damage to AAV.

In conclusion, the present study indicates that SNPs observed in *AKT1* (rs2498786, rs2494752 and rs5811155) are associated with MPA risk in a Chinese population. SNPs in *AKT1* (rs2498786, rs2494752 and rs5811155) may affect MPA risk by impact on the expression of AKT1 in neutrophils to regulate the formation of ANCA, NETs, and DCs and macrophages to regulate their functions. However, this hypothesis requires further clinical validation and experimental confirmation to provide precision medicine for MPA/AAV. This study was a single-center study, and because of the low prevalence rate of MPA, the sample size was too small to divide into the discovery and replication cohort of patients.

## Supplementary Information

Below is the link to the electronic supplementary material.

Supplementary file1 (DOCX 158 KB)

Supplementary file2 (DOCX 13 KB)

Supplementary file3 Supplement Figure 1 Summaries of functionality scores for rs2498786, rs2494752, and rs5811155. Note: This figure illustrates the annotation of rs2498786, rs2494752, and rs5811155. Data were obtained from the 3DSNP website (omic.tech). Abbreviations: 3D gene, 3D interacting genes. Enhancer, Enhancer state. Promoter, Promoter state. Tfbs, transcription factor binding sites. Motif, sequence motifs (EPS 1312 KB)

Supplementary file4 Supplement Figure 2 Integrated regulation from ENCODE. Note: In cell lines derived from blood or vascular tissue, four histone modifications (CTCF, H3K4Me1, H3K4Me3, and H3K27Ac) associated with gene regulation and the three loci are shown in part A. The values of histone modifications vary from 0 to 6.48. Information about transcription factor binding related to the three loci is shown in Figs. B. Transcription factors that can bind to two or more regions are color-identified. DNase I sensitivity associated with gene regulation and the three loci are shown in part C. The values of DNase I signal vary from 0.28 to 14.37 in cell lines derived from blood or vascular tissue. Abbreviations: B cells, B cells CD20+. Monocytes, Monocytes CD14+ RO01746. (EPS 1588 KB)

Supplementary file5 Supplement Figure 3 Expression quantitative trait locus (eQTL) of rs2498786, rs2494752, and rs5811155. Note: Data were obtained from the 3DSNP website (omic.tech). AKT1 regulates blood tissue, and loci (rs2498786, rs2494752, and rs5811155) modulate vascular tissue by affecting SIVA1. Abbreviations: a, the effect size of rs2498786, rs2494752, and rs5811155 on whole blood by AKT1. b, the effect size of rs2498786 on whole blood by SIVA1. c, the effect size of rs2498786 on artery aorta by SIVA1. d, the effect size of rs2498786 on artery tibial by SIVA1. e, the effect size of rs5811155 on artery aorta by SIVA1. f, the effect size of rs2494752 on artery aorta by SIVA1. (EPS 391 KB)

## Data Availability

Data available on request from the authors.
