# Supplementary material for: Association between the AKT1 single nucleotide polymorphism (rs2498786, rs2494752 and rs5811155) and microscopic polyangiitis risk in a Chinese population
Source: Mol Genet Genomics. 2023 Apr 7;298(3):767–76. doi: 10.1007/s00438-023-02012-6 (PMC10133348; doi:10.1007/s00438-023-02012-6)
Supplement: Supplementary file 1 — Supplementary file1 (DOCX 158 KB) [file 438_2023_2012_MOESM1_ESM.docx]

1. **Additional descriptive data**

**Table 1** Distribution of alleles (rs2498786, rs2494752 and rs5811155) in AKT1 in 1000Genomes and gnomAD-Genomes among races (East asian, African, American and Europe)

|  | Vriant Type | Project | Race | Ref | Alt |
| --- | --- | --- | --- | --- | --- |
| rs2498786 | SNV  C>G | 1000Genomes | Europe | 0.09 | 0.91 |
|  |  |  | American | 0.24 | 0.76 |
|  |  |  | African | 0.24 | 0.76 |
|  |  |  | East asian | 0.67 | 0.33 |
|  |  | gnomAD-Genomes | Europe | 0.08 | 0.92 |
|  |  |  | American | 0.22 | 0.78 |
|  |  |  | African | 0.20 | 0.80 |
|  |  |  | East asian | 0.68 | 0.32 |
| rs2494752 | SNV  A>G | 1000Genomes | Europe | 0.09 | 0.91 |
|  |  |  | American | 0.24 | 0.76 |
|  |  |  | African | 0.24 | 0.76 |
|  |  |  | East asian | 0.71 | 0.29 |
|  |  | gnomAD-Genomes | Europe | 0.08 | 0.92 |
|  |  |  | American | 0.22 | 0.78 |
|  |  |  | African | 0.20 | 0.80 |
|  |  |  | East asian | 0.71 | 0.29 |
| rs5811155 | Insertion  insT | 1000Genomes | Europe | 0.39 | 0.61 |
|  |  |  | American | 0.52 | 0.48 |
|  |  |  | African | 0.75 | 0.25 |
|  |  |  | East asian | 0.79 | 0.21 |
|  |  | gnomAD-Genomes | Europe | 0.39 | 0.61 |
|  |  |  | American | 0.49 | 0.51 |
|  |  |  | African | 0.69 | 0.31 |
|  |  |  | East asian | 0.80 | 0.20 |

Note: Data of 1000Genomes and gnomAD-Genomes were obtainde from public database (NCBI).

**Table 2** Information of alleles in AKT1 (MPA Group, n=208, Guangxi, n=208, 1000Genomes, n=387)

| Loci | Vriant Type | Group | Ref | Alt | MAF | HWE(p-value) |
| --- | --- | --- | --- | --- | --- | --- |
| rs2498786 | SNV  C>G | MPA Group | 355(85%) | 61(15%) | 0.15 | 0.58 |
|  |  | Guangxi | 319(77%) | 97(23%) | 0.23 | 0.051 |
|  |  | 1000Genomes | 598(77%) | 176(23%) | 0.23 | - |
| rs2494752 | SNV  A>G | MPA Group | 325(78%) | 91(22%) | 0.22 | 0.42 |
|  |  | Guangxi | 291(70%) | 125(30%) | 0.30 | 0.87 |
|  |  | 1000Genomes | 511(66%) | 263(34%) | 0.34 | - |
| rs5811155 | Insertion  insT | MPA Group | - | 94(23%) | 0.23 | 0.56 |
|  |  | Guangxi | - | 132(32%) | 0.32 | 0.87 |
|  |  | 1000Genomes | - | 285(37%) | 0.37 | - |

Note: The HEW is calculated by Pearson’s chi-squared test on SNPStats ([web tool for SNP analysis](https://www.snpstats.net/start.htm)).

Abbreviations: SNV, Single Nucleotide Variation. Ref, Ref Allele. Alt, Alt Allele. MAF, Minor Allele Frequency. HWE, Hardy–Weinberg equilibrium.

**Table 3:** Association of Genotypes in AKT1 with the risk of MPA (n=416, adjusted by sex) (without data in 1000Genomes)

| Loci | Model | Genotype | Guangxi  (n=208) | MPA Group (n=208) | OR (95% CI) | P-value |
| --- | --- | --- | --- | --- | --- | --- |
| rs2498786 | Codominant | CC | 117 (56.2%) | 150 (72.1%) | 1.00 | **0.0032** |
|  |  | C/G | 85 (40.9%) | 55 (26.4%) | 0.51 (0.33-0.77) |  |
|  |  | G/G | 6 (2.9%) | 3 (1.4%) | 0.39 (0.10-1.60) |  |
|  | Dominant | CC | 117 (56.2%) | 150 (72.1%) | 1.00 |  |
|  |  | C/G-G/G | 91 (43.8%) | 58 (27.9%) | 0.50 (0.33-0.75) | **0.0007** |
|  | Recessive | C/C-C/G | 202 (97.1%) | 205 (98.6%) | 1.00 |  |
|  |  | G/G | 6 (2.9%) | 3 (1.4%) | 0.50 (0.12-2.02) | 0.3100 |
|  | Overdominant | C/C-G/G | 123 (59.1%) | 153 (73.6%) | 1.00 |  |
|  |  | C/G | 85 (40.9%) | 55 (26.4%) | 0.52 (0.34-0.79) | **0.0019** |
| rs2494752 | Codominant | A/A | 101 (48.6%) | 129 (62%) | 1.00 | **0.0210** |
|  |  | A/G | 89 (42.8%) | 67 (32.2%) | 0.59 (0.39-0.89) |  |
|  |  | G/G | 18 (8.7%) | 12 (5.8%) | 0.52 (0.24-1.14) |  |
|  | Dominant | A/A | 101 (48.6%) | 129 (62%) | 1.00 |  |
|  |  | A/G-G/G | 107 (51.4%) | 79 (38%) | 0.58 (0.39-0.86) | **0.0059** |
|  | Recessive | A/A-A/G | 190 (91.3%) | 196 (94.2%) | 1.00 |  |
|  |  | G/G | 18 (8.7%) | 12 (5.8%) | 0.65 (0.30-1.38) | 0.2600 |
|  | Overdominant | A/A-G/G | 119 (57.2%) | 141 (67.8%) | 1.00 |  |
|  |  | A/G | 89 (42.8%) | 67 (32.2%) | 0.64 (0.43-0.95) | **0.0260** |
| rs5811155 | Codominant | C/C | 96 (46.1%) | 126 (60.6%) | 1.00 |  |
|  |  | C/T | 92 (44.2%) | 70 (33.6%) | 0.58 (0.39-0.87) | **0.0110** |
|  |  | T/T | 20 (9.6%) | 12 (5.8%) | 0.46 (0.21-0.98) |  |
|  | Dominant | C/C | 96 (46.1%) | 126 (60.6%) | 1.00 |  |
|  |  | C/T-T/T | 112 (53.9%) | 82 (39.4%) | 0.56 (0.38-0.82) | **0.0032** |
|  | Recessive | C/C- C/T | 188 (90.4%) | 196 (94.2%) | 1.00 |  |
|  |  | T/T | 20 (9.6%) | 12 (5.8%) | 0.58 (0.27-1.21) | 0.1400 |
|  | Overdominant | C/C-T/T | 116 (55.8%) | 138 (66.3%) | 1.00 |  |
|  |  | C/T | 92 (44.2%) | 70 (33.6%) | 0.64 (0.43-0.95) | **0.0270** |

Note: Data were calculated on SNPStats ([web tool for SNP analysis](https://www.snpstats.net/start.htm)).

1. Analysis Results on tool SNPStats
   1. Association between AKT1 and the risk of MPA

| SNPStats results | |
| --- | --- |
| **Index** |  |
| \| [**Descriptive statistics**](https://www.snpstats.net/analyzer.php#covdesc) \| \| \| --- \| --- \| \| [**Single-SNP analysis**](https://www.snpstats.net/analyzer.php#singlesnp) \| \| \|  \| [rs2498786](https://www.snpstats.net/analyzer.php#snp1) \| \|  \| [rs2494752](https://www.snpstats.net/analyzer.php#snp2) \| \|  \| [rs5811155](https://www.snpstats.net/analyzer.php#snp3) \| \| [**Multiple-SNP analysis**](https://www.snpstats.net/analyzer.php#multiplesnp) \| \| \|  \| [Linkage disequilibrium analysis](https://www.snpstats.net/analyzer.php#ldanalysis) \| \|  \| [Haplotype analysis](https://www.snpstats.net/analyzer.php#haploanalysis) \| |  |
| **Descriptive statistics** |  |
| **Response variable:**status **Type:**categorical |  |
| \|  \| **n** \| **missing** \| **unique** \| \| --- \| --- \| --- \| --- \| \| **All subjects** \| 803 \| 0 \| 2 \| \| **status=Ca** \| 595 (74.1%) \| --- \| --- \| \| **status=Co** \| 208 (25.9%) \| --- \| --- \| |  |
| **Covariate:**sex **Type:**categorical |  |
| \|  \| **n** \| **missing** \| **unique** \| \| --- \| --- \| --- \| --- \| \| **All subjects** \| 803 \| 0 \| 2 \| \| **status=Ca** \| 595 \| 0 \| 2 \| \| **status=Co** \| 208 \| 0 \| 2 \|  \|  \| **female** \| **male** \| \| --- \| --- \| --- \| \| **All subjects** \| 460 (57%) \| 343 (43%) \| \| **status=Ca** \| 330 (55%) \| 265 (45%) \| \| **status=Co** \| 130 (62%) \| 78 (38%) \| |  |
| **Single-SNP analysis** |  |
| **SNP:**rs2498786 |  |
| **Percentage of typed samples:**803/803 (100%)   \| **rs2498786 allele frequencies (n=803)** \| \| \| \| \| \| \| \| --- \| --- \| --- \| --- \| --- \| --- \| --- \| \|  \| **All subjects** \| \| **status=Ca** \| \| **status=Co** \| \| \| **Allele** \| **Count** \| **Proportion** \| **Count** \| **Proportion** \| **Count** \| **Proportion** \| \| C \| 1272 \| 0.79 \| 917 \| 0.77 \| 355 \| 0.85 \| \| G \| 334 \| 0.21 \| 273 \| 0.23 \| 61 \| 0.15 \|  \| **rs2498786 genotype frequencies (n=803)** \| \| \| \| \| \| \| \| --- \| --- \| --- \| --- \| --- \| --- \| --- \| \|  \| **All subjects** \| \| **status=Ca** \| \| **status=Co** \| \| \| **Genotype** \| **Count** \| **Proportion** \| **Count** \| **Proportion** \| **Count** \| **Proportion** \| \| C/C \| 506 \| 0.63 \| 356 \| 0.6 \| 150 \| 0.72 \| \| C/G \| 260 \| 0.32 \| 205 \| 0.34 \| 55 \| 0.26 \| \| G/G \| 37 \| 0.05 \| 34 \| 0.06 \| 3 \| 0.01 \|  \| **rs2498786 exact test for Hardy-Weinberg equilibrium (n=803)** \| \| \| \| \| \| \| \| --- \| --- \| --- \| --- \| --- \| --- \| --- \| \|  \| **N11** \| **N12** \| **N22** \| **N1** \| **N2** \| **P-value** \| \| **All subjects** \| 506 \| 260 \| 37 \| 1272 \| 334 \| 0.59 \| \| **status=Ca** \| 356 \| 205 \| 34 \| 917 \| 273 \| 0.56 \| \| **status=Co** \| 150 \| 55 \| 3 \| 355 \| 61 \| 0.58 \|  \| **rs2498786 association with response status (n=803, adjusted by sex)** \| \| \| \| \| \| \| \| \| --- \| --- \| --- \| --- \| --- \| --- \| --- \| --- \| \| **Model** \| **Genotype** \| **status=Ca** \| **status=Co** \| **OR (95% CI)** \| **P-value** \| **AIC** \| **BIC** \| \| Codominant \| C/C \| 356 (59.8%) \| 150 (72.1%) \| 1.00 \| 7e-04 \| 908.9 \| 927.7 \| \| C/G \| 205 (34.5%) \| 55 (26.4%) \| **0.63 (0.44-0.90)** \| \| G/G \| 34 (5.7%) \| 3 (1.4%) \| **0.21 (0.06-0.69)** \| \| Dominant \| C/C \| 356 (59.8%) \| 150 (72.1%) \| 1.00 \| 0.0012 \| 911.1 \| 925.1 \| \| C/G-G/G \| 239 (40.2%) \| 58 (27.9%) \| **0.57 (0.41-0.81)** \| \| Recessive \| C/C-C/G \| 561 (94.3%) \| 205 (98.6%) \| 1.00 \| 0.0048 \| 913.6 \| 927.6 \| \| G/G \| 34 (5.7%) \| 3 (1.4%) \| **0.24 (0.07-0.79)** \| \| Overdominant \| C/C-G/G \| 390 (65.5%) \| 153 (73.6%) \| 1.00 \| 0.029 \| 916.8 \| 930.8 \| \| C/G \| 205 (34.5%) \| 55 (26.4%) \| **0.68 (0.48-0.97)** \| \| Log-additive \| --- \| --- \| --- \| **0.58 (0.42-0.78)** \| 2e-04 \| 907.9 \| 922 \|  \| **Interaction analysis with covariate sex** \| \| --- \|  \| **rs2498786 and sex cross-classification interaction table (n=803, crude analysis)** \| \| \| \| \| \| \| \| --- \| --- \| --- \| --- \| --- \| --- \| --- \| \|  \| **female** \| \| \| **male** \| \| \| \|  \| **status=Ca** \| **status=Co** \| **OR (95% CI)** \| **status=Ca** \| **status=Co** \| **OR (95% CI)** \| \| **C/C** \| 186 \| 101 \| 1.00 \| 170 \| 49 \| **0.53 (0.36-0.79)** \| \| **C/G** \| 125 \| 27 \| **0.40 (0.25-0.64)** \| 80 \| 28 \| 0.64 (0.39-1.06) \| \| **G/G** \| 19 \| 2 \| **0.19 (0.04-0.85)** \| 15 \| 1 \| **0.12 (0.02-0.94)** \| \| **Interaction p-value: 0.0097** \| \| \| \| \| \| \|  \| **sex within rs2498786 (n=803, crude analysis)** \| \| \| --- \| --- \| \| **C/C** \| \|  \| **status=Ca** \| **status=Co** \| **OR (95% CI)** \| \| --- \| --- \| --- \| --- \| \| **female** \| 186 \| 101 \| 1.00 \| \| **male** \| 170 \| 49 \| **0.53 (0.36-0.79)** \| \| \| **C/G** \| \|  \| **status=Ca** \| **status=Co** \| **OR (95% CI)** \| \| --- \| --- \| --- \| --- \| \| **female** \| 125 \| 27 \| 1.00 \| \| **male** \| 80 \| 28 \| 1.62 (0.89-2.95) \| \| \| **G/G** \| \|  \| **status=Ca** \| **status=Co** \| **OR (95% CI)** \| \| --- \| --- \| --- \| --- \| \| **female** \| 19 \| 2 \| 1.00 \| \| **male** \| 15 \| 1 \| 0.63 (0.05-7.67) \| \| \| **Test for interaction in the trend: 0.011** \| \|  \| **rs2498786 within sex (n=803, crude analysis)** \| \| \| --- \| --- \| \| **female** \| \|  \| **status=Ca** \| **status=Co** \| **OR (95% CI)** \| \| --- \| --- \| --- \| --- \| \| **C/C** \| 186 \| 101 \| 1.00 \| \| **C/G** \| 125 \| 27 \| **0.40 (0.25-0.64)** \| \| **G/G** \| 19 \| 2 \| **0.19 (0.04-0.85)** \| \| \| **male** \| \|  \| **status=Ca** \| **status=Co** \| **OR (95% CI)** \| \| --- \| --- \| --- \| --- \| \| **C/C** \| 170 \| 49 \| 1.00 \| \| **C/G** \| 80 \| 28 \| 1.21 (0.71-2.07) \| \| **G/G** \| 15 \| 1 \| 0.23 (0.03-1.79) \| \| \| **Test for interaction in the trend: 0.0097** \| \| |  |
| **SNP:**rs2494752 |  |
| **Percentage of typed samples:**803/803 (100%)   \| **rs2494752 allele frequencies (n=803)** \| \| \| \| \| \| \| \| --- \| --- \| --- \| --- \| --- \| --- \| --- \| \|  \| **All subjects** \| \| **status=Ca** \| \| **status=Co** \| \| \| **Allele** \| **Count** \| **Proportion** \| **Count** \| **Proportion** \| **Count** \| **Proportion** \| \| A \| 1127 \| 0.7 \| 802 \| 0.67 \| 325 \| 0.78 \| \| G \| 479 \| 0.3 \| 388 \| 0.33 \| 91 \| 0.22 \|  \| **rs2494752 genotype frequencies (n=803)** \| \| \| \| \| \| \| \| --- \| --- \| --- \| --- \| --- \| --- \| --- \| \|  \| **All subjects** \| \| **status=Ca** \| \| **status=Co** \| \| \| **Genotype** \| **Count** \| **Proportion** \| **Count** \| **Proportion** \| **Count** \| **Proportion** \| \| A/A \| 409 \| 0.51 \| 280 \| 0.47 \| 129 \| 0.62 \| \| A/G \| 309 \| 0.38 \| 242 \| 0.41 \| 67 \| 0.32 \| \| G/G \| 85 \| 0.11 \| 73 \| 0.12 \| 12 \| 0.06 \|  \| **rs2494752 exact test for Hardy-Weinberg equilibrium (n=803)** \| \| \| \| \| \| \| \| --- \| --- \| --- \| --- \| --- \| --- \| --- \| \|  \| **N11** \| **N12** \| **N22** \| **N1** \| **N2** \| **P-value** \| \| **All subjects** \| 409 \| 309 \| 85 \| 1127 \| 479 \| **0.023** \| \| **status=Ca** \| 280 \| 242 \| 73 \| 802 \| 388 \| 0.076 \| \| **status=Co** \| 129 \| 67 \| 12 \| 325 \| 91 \| 0.42 \|  \| **rs2494752 association with response status (n=803, adjusted by sex)** \| \| \| \| \| \| \| \| \| --- \| --- \| --- \| --- \| --- \| --- \| --- \| --- \| \| **Model** \| **Genotype** \| **status=Ca** \| **status=Co** \| **OR (95% CI)** \| **P-value** \| **AIC** \| **BIC** \| \| Codominant \| A/A \| 280 (47.1%) \| 129 (62%) \| 1.00 \| 3e-04 \| 907.4 \| 926.2 \| \| A/G \| 242 (40.7%) \| 67 (32.2%) \| **0.60 (0.43-0.85)** \| \| G/G \| 73 (12.3%) \| 12 (5.8%) \| **0.36 (0.19-0.68)** \| \| Dominant \| A/A \| 280 (47.1%) \| 129 (62%) \| 1.00 \| 2e-04 \| 907.9 \| 922 \| \| A/G-G/G \| 315 (52.9%) \| 79 (38%) \| **0.55 (0.40-0.76)** \| \| Recessive \| A/A-A/G \| 522 (87.7%) \| 196 (94.2%) \| 1.00 \| 0.0058 \| 913.9 \| 928 \| \| G/G \| 73 (12.3%) \| 12 (5.8%) \| **0.44 (0.23-0.83)** \| \| Overdominant \| A/A-G/G \| 353 (59.3%) \| 141 (67.8%) \| 1.00 \| 0.032 \| 917 \| 931 \| \| A/G \| 242 (40.7%) \| 67 (32.2%) \| **0.70 (0.50-0.97)** \| \| Log-additive \| --- \| --- \| --- \| **0.60 (0.47-0.78)** \| 1e-04 \| 905.4 \| 919.5 \|  \| **Interaction analysis with covariate sex** \| \| --- \|  \| **rs2494752 and sex cross-classification interaction table (n=803, crude analysis)** \| \| \| \| \| \| \| \| --- \| --- \| --- \| --- \| --- \| --- \| --- \| \|  \| **female** \| \| \| **male** \| \| \| \|  \| **status=Ca** \| **status=Co** \| **OR (95% CI)** \| **status=Ca** \| **status=Co** \| **OR (95% CI)** \| \| **A/A** \| 155 \| 85 \| 1.00 \| 125 \| 44 \| **0.64 (0.42-0.99)** \| \| **A/G** \| 133 \| 40 \| **0.55 (0.35-0.85)** \| 109 \| 27 \| **0.45 (0.27-0.74)** \| \| **G/G** \| 42 \| 5 \| **0.22 (0.08-0.57)** \| 31 \| 7 \| **0.41 (0.17-0.97)** \| \| **Interaction p-value:**0.25 \| \| \| \| \| \| \|  \| **sex within rs2494752 (n=803, crude analysis)** \| \| \| --- \| --- \| \| **A/A** \| \|  \| **status=Ca** \| **status=Co** \| **OR (95% CI)** \| \| --- \| --- \| --- \| --- \| \| **female** \| 155 \| 85 \| 1.00 \| \| **male** \| 125 \| 44 \| **0.64 (0.42-0.99)** \| \| \| **A/G** \| \|  \| **status=Ca** \| **status=Co** \| **OR (95% CI)** \| \| --- \| --- \| --- \| --- \| \| **female** \| 133 \| 40 \| 1.00 \| \| **male** \| 109 \| 27 \| 0.82 (0.48-1.43) \| \| \| **G/G** \| \|  \| **status=Ca** \| **status=Co** \| **OR (95% CI)** \| \| --- \| --- \| --- \| --- \| \| **female** \| 42 \| 5 \| 1.00 \| \| **male** \| 31 \| 7 \| 1.90 (0.55-6.54) \| \| \| **Test for interaction in the trend:**0.13 \| \|  \| **rs2494752 within sex (n=803, crude analysis)** \| \| \| --- \| --- \| \| **female** \| \|  \| **status=Ca** \| **status=Co** \| **OR (95% CI)** \| \| --- \| --- \| --- \| --- \| \| **A/A** \| 155 \| 85 \| 1.00 \| \| **A/G** \| 133 \| 40 \| **0.55 (0.35-0.85)** \| \| **G/G** \| 42 \| 5 \| **0.22 (0.08-0.57)** \| \| \| **male** \| \|  \| **status=Ca** \| **status=Co** \| **OR (95% CI)** \| \| --- \| --- \| --- \| --- \| \| **A/A** \| 125 \| 44 \| 1.00 \| \| **A/G** \| 109 \| 27 \| 0.70 (0.41-1.21) \| \| **G/G** \| 31 \| 7 \| 0.64 (0.26-1.56) \| \| \| **Test for interaction in the trend:**0.25 \| \| |  |
| **SNP:**rs5811155 |  |
| **Percentage of typed samples:**803/803 (100%)   \| **rs5811155 allele frequencies (n=803)** \| \| \| \| \| \| \| \| --- \| --- \| --- \| --- \| --- \| --- \| --- \| \|  \| **All subjects** \| \| **status=Ca** \| \| **status=Co** \| \| \| **Allele** \| **Count** \| **Proportion** \| **Count** \| **Proportion** \| **Count** \| **Proportion** \| \| C \| 1095 \| 0.68 \| 773 \| 0.65 \| 322 \| 0.77 \| \| T \| 511 \| 0.32 \| 417 \| 0.35 \| 94 \| 0.23 \|  \| **rs5811155 genotype frequencies (n=803)** \| \| \| \| \| \| \| \| --- \| --- \| --- \| --- \| --- \| --- \| --- \| \|  \| **All subjects** \| \| **status=Ca** \| \| **status=Co** \| \| \| **Genotype** \| **Count** \| **Proportion** \| **Count** \| **Proportion** \| **Count** \| **Proportion** \| \| C/C \| 386 \| 0.48 \| 260 \| 0.44 \| 126 \| 0.61 \| \| C/T \| 323 \| 0.4 \| 253 \| 0.43 \| 70 \| 0.34 \| \| T/T \| 94 \| 0.12 \| 82 \| 0.14 \| 12 \| 0.06 \|  \| **rs5811155 exact test for Hardy-Weinberg equilibrium (n=803)** \| \| \| \| \| \| \| \| --- \| --- \| --- \| --- \| --- \| --- \| --- \| \|  \| **N11** \| **N12** \| **N22** \| **N1** \| **N2** \| **P-value** \| \| **All subjects** \| 386 \| 323 \| 94 \| 1095 \| 511 \| **0.042** \| \| **status=Ca** \| 260 \| 253 \| 82 \| 773 \| 417 \| 0.11 \| \| **status=Co** \| 126 \| 70 \| 12 \| 322 \| 94 \| 0.56 \|  \| **rs5811155 association with response status (n=803, adjusted by sex)** \| \| \| \| \| \| \| \| \| --- \| --- \| --- \| --- \| --- \| --- \| --- \| --- \| \| **Model** \| **Genotype** \| **status=Ca** \| **status=Co** \| **OR (95% CI)** \| **P-value** \| **AIC** \| **BIC** \| \| Codominant \| C/C \| 260 (43.7%) \| 126 (60.6%) \| 1.00 \| <0.0001 \| 902.3 \| 921 \| \| C/T \| 253 (42.5%) \| 70 (33.6%) \| **0.57 (0.41-0.80)** \| \| T/T \| 82 (13.8%) \| 12 (5.8%) \| **0.31 (0.16-0.58)** \| \| Dominant \| C/C \| 260 (43.7%) \| 126 (60.6%) \| 1.00 \| <0.0001 \| 904.1 \| 918.2 \| \| C/T-T/T \| 335 (56.3%) \| 82 (39.4%) \| **0.51 (0.37-0.70)** \| \| Recessive \| C/C-C/T \| 513 (86.2%) \| 196 (94.2%) \| 1.00 \| 0.0011 \| 910.9 \| 925 \| \| T/T \| 82 (13.8%) \| 12 (5.8%) \| **0.39 (0.21-0.73)** \| \| Overdominant \| C/C-T/T \| 342 (57.5%) \| 138 (66.3%) \| 1.00 \| 0.024 \| 916.4 \| 930.5 \| \| C/T \| 253 (42.5%) \| 70 (33.6%) \| **0.68 (0.49-0.95)** \| \| Log-additive \| --- \| --- \| --- \| **0.56 (0.43-0.72)** \| <0.0001 \| 900.3 \| 914.4 \|  \| **Interaction analysis with covariate sex** \| \| --- \|  \| **rs5811155 and sex cross-classification interaction table (n=803, crude analysis)** \| \| \| \| \| \| \| \| --- \| --- \| --- \| --- \| --- \| --- \| --- \| \|  \| **female** \| \| \| **male** \| \| \| \|  \| **status=Ca** \| **status=Co** \| **OR (95% CI)** \| **status=Ca** \| **status=Co** \| **OR (95% CI)** \| \| **C/C** \| 141 \| 84 \| 1.00 \| 119 \| 42 \| **0.59 (0.38-0.92)** \| \| **C/T** \| 144 \| 41 \| **0.48 (0.31-0.74)** \| 109 \| 29 \| **0.45 (0.27-0.73)** \| \| **T/T** \| 45 \| 5 \| **0.19 (0.07-0.49)** \| 37 \| 7 \| **0.32 (0.14-0.74)** \| \| **Interaction p-value:**0.18 \| \| \| \| \| \| \|  \| **sex within rs5811155 (n=803, crude analysis)** \| \| \| --- \| --- \| \| **C/C** \| \|  \| **status=Ca** \| **status=Co** \| **OR (95% CI)** \| \| --- \| --- \| --- \| --- \| \| **female** \| 141 \| 84 \| 1.00 \| \| **male** \| 119 \| 42 \| **0.59 (0.38-0.92)** \| \| \| **C/T** \| \|  \| **status=Ca** \| **status=Co** \| **OR (95% CI)** \| \| --- \| --- \| --- \| --- \| \| **female** \| 144 \| 41 \| 1.00 \| \| **male** \| 109 \| 29 \| 0.93 (0.55-1.60) \| \| \| **T/T** \| \|  \| **status=Ca** \| **status=Co** \| **OR (95% CI)** \| \| --- \| --- \| --- \| --- \| \| **female** \| 45 \| 5 \| 1.00 \| \| **male** \| 37 \| 7 \| 1.70 (0.50-5.81) \| \| \| **Test for interaction in the trend:**0.066 \| \|  \| **rs5811155 within sex (n=803, crude analysis)** \| \| \| --- \| --- \| \| **female** \| \|  \| **status=Ca** \| **status=Co** \| **OR (95% CI)** \| \| --- \| --- \| --- \| --- \| \| **C/C** \| 141 \| 84 \| 1.00 \| \| **C/T** \| 144 \| 41 \| **0.48 (0.31-0.74)** \| \| **T/T** \| 45 \| 5 \| **0.19 (0.07-0.49)** \| \| \| **male** \| \|  \| **status=Ca** \| **status=Co** \| **OR (95% CI)** \| \| --- \| --- \| --- \| --- \| \| **C/C** \| 119 \| 42 \| 1.00 \| \| **C/T** \| 109 \| 29 \| 0.75 (0.44-1.29) \| \| **T/T** \| 37 \| 7 \| 0.54 (0.22-1.29) \| \| \| **Test for interaction in the trend:**0.18 \| \| |  |
| **Multiple-SNP analysis** |  |
| \| **Linkage disequilibrium analysis** \| \| --- \|   **D statistic**   \|  \| rs2498786 \| rs2494752 \| rs5811155 \| \| --- \| --- \| --- \| --- \| \| rs2498786 \| . \| 0.1265 \| 0.1417 \| \| rs2494752 \| . \| . \| 0.2033 \| \| rs5811155 \| . \| . \| . \|   **D' statistic**   \|  \| rs2498786 \| rs2494752 \| rs5811155 \| \| --- \| --- \| --- \| --- \| \| rs2498786 \| . \| 0.8665 \| 0.9996 \| \| rs2494752 \| . \| . \| 0.9998 \| \| rs5811155 \| . \| . \| . \|   **r statistic**   \|  \| rs2498786 \| rs2494752 \| rs5811155 \| \| --- \| --- \| --- \| --- \| \| rs2498786 \| . \| 0.6811 \| 0.7498 \| \| rs2494752 \| . \| . \| 0.9541 \| \| rs5811155 \| . \| . \| . \|   **P-values**   \|  \| rs2498786 \| rs2494752 \| rs5811155 \| \| --- \| --- \| --- \| --- \| \| rs2498786 \| . \| 0 \| 0 \| \| rs2494752 \| . \| . \| 0 \| \| rs5811155 \| . \| . \| . \| |  |
| \| **Haplotype analysis** \| \| --- \| |  |
| \| **Haplotype frequencies estimation (n=803)** \| \| \| \| \| \| \| \| \| --- \| --- \| --- \| --- \| --- \| --- \| --- \| --- \| \|  \| **rs2498786** \| **rs2494752** \| **rs5811155** \| **Total** \| **group.Ca** \| **group.Co** \| **Cumulative frequency** \| \| 1 \| C \| A \| C \| 0.6818 \| 0.6496 \| 0.774 \| 0.6818 \| \| 2 \| G \| G \| T \| 0.188 \| 0.205 \| 0.1394 \| 0.8699 \| \| 3 \| C \| G \| T \| 0.1102 \| 0.121 \| 0.0793 \| 0.9801 \| \| 4 \| G \| A \| T \| 0.0199 \| 0.0244 \| 0.0072 \| 1 \| \| 5 \| C \| A \| T \| 0 \| 0 \| NA \| 1 \| |  |
| \| **Haplotype association with response (n=803, adjusted by sex)** \| \| \| \| \| \| \| \| \| \| --- \| --- \| --- \| --- \| --- \| --- \| --- \| --- \| --- \| \|  \| **rs2498786** \| **rs2494752** \| **rs5811155** \| **Freq** \| **OR (95% CI)** \| **P-value** \|  \|  \| \| 1 \| C \| A \| C \| 0.6818 \| 1.00 \| --- \|  \|  \| \| 2 \| G \| G \| T \| 0.188 \| **0.58 (0.42 - 0.79)** \| 7e-04 \|  \|  \| \| 3 \| C \| G \| T \| 0.1102 \| **0.59 (0.40 - 0.87)** \| 0.0083 \|  \|  \| \| 4 \| G \| A \| T \| 0.0199 \| **0.25 (0.08 - 0.82)** \| 0.023 \|  \|  \| \| **Global haplotype association p-value: <0.0001** \| \| \| \| \| \| \| \| \| |  |
| \| **Haplotype interaction analysis with covariate sex** \| \| --- \| |  |
| \| **Haplotype and sex cross-classification interaction table (n=803, crude analysis)** \| \| \| \| \| --- \| --- \| --- \| --- \| \|  \|  \| **female** \| **male** \| \| **Haplotype** \| **Frequency** \| **OR (95% CI)** \| **OR (95% CI)** \| \| **CAC** \| 0.6818 \| 1.00 \| **0.58 (0.38 - 0.89)** \| \| **CGT** \| 0.1102 \| 0.61 (0.36 - 1.02) \| **0.34 (0.18 - 0.62)** \| \| **GAT** \| 0.0199 \| **0.10 (0.01 - 0.79)** \| 0.32 (0.07 - 1.36) \| \| **GGT** \| 0.188 \| **0.42 (0.28 - 0.65)** \| **0.52 (0.32 - 0.84)** \| \| **Interaction p-value:**0.069 \| \| \| \| |  |
| \| **Haplotypes within sex (n=803, crude analysis)** \| \| \| \| \| --- \| --- \| --- \| --- \| \|  \|  \| **female** \| **male** \| \| **Haplotype** \| **Frequency** \| **OR (95% CI)** \| **OR (95% CI)** \| \| **CAC** \| 0.6818 \| 1.00 \| 1.00 \| \| **CGT** \| 0.1102 \| 0.61 (0.36 - 1.02) \| 0.58 (0.32 - 1.06) \| \| **GAT** \| 0.0199 \| **0.10 (0.01 - 0.79)** \| 0.54 (0.13 - 2.32) \| \| **GGT** \| 0.188 \| **0.42 (0.28 - 0.65)** \| 0.90 (0.56 - 1.44) \| |  |
| \| **sex within haplotypes (n=803, crude analysis)** \| \| \| \| \| --- \| --- \| --- \| --- \| \|  \|  \| **female** \| **male** \| \| **Haplotype** \| **Frequency** \| **OR (95% CI)** \| **OR (95% CI)** \| \| **CAC** \| 0.6818 \| 1.00 \| **0.58 (0.38 - 0.89)** \| \| **CGT** \| 0.1102 \| 1.00 \| 0.56 (0.27 - 1.17) \| \| **GAT** \| 0.0199 \| 1.00 \| 3.04 (0.25 - 36.23) \| \| **GGT** \| 0.188 \| 1.00 \| 1.23 (0.71 - 2.13) \| |  |

1. Association between AKT1 and MPO-ANCA + in MPA patients

| SNPStats results | |
| --- | --- |
| **Index** |  |
| \| **[Descriptive statistics](https://www.snpstats.net/analyzer.php#covdesc)** \| \| \| --- \| --- \| \| **[Single-SNP analysis](https://www.snpstats.net/analyzer.php#singlesnp)** \| \| \|  \| [rs2498786](https://www.snpstats.net/analyzer.php#snp1) \| \|  \| [rs2494752](https://www.snpstats.net/analyzer.php#snp2) \| \|  \| [rs5811155](https://www.snpstats.net/analyzer.php#snp3) \| \| **[Multiple-SNP analysis](https://www.snpstats.net/analyzer.php#multiplesnp)** \| \| \|  \| [Linkage disequilibrium analysis](https://www.snpstats.net/analyzer.php#ldanalysis) \| \|  \| [Haplotype analysis](https://www.snpstats.net/analyzer.php#haploanalysis) \| |  |
| **Descriptive statistics** |  |
| **Response variable:**status **Type:**categorical |  |
| \|  \| **n** \| **missing** \| **unique** \| \| --- \| --- \| --- \| --- \| \| **All subjects** \| 736 \| 0 \| 2 \| \| **status=Ca** \| 595 (80.84%) \| --- \| --- \| \| **status=Co** \| 141 (19.16%) \| --- \| --- \| |  |
| **Covariate:**sex **Type:**categorical |  |
| \|  \| **n** \| **missing** \| **unique** \| \| --- \| --- \| --- \| --- \| \| **All subjects** \| 736 \| 0 \| 2 \| \| **status=Ca** \| 595 \| 0 \| 2 \| \| **status=Co** \| 141 \| 0 \| 2 \|  \|  \| **female** \| **male** \| \| --- \| --- \| --- \| \| **All subjects** \| 422 (57%) \| 314 (43%) \| \| **status=Ca** \| 330 (55%) \| 265 (45%) \| \| **status=Co** \| 92 (65%) \| 49 (35%) \| |  |
| **Single-SNP analysis** |  |
| **SNP:**rs2498786 |  |
| **Percentage of typed samples:**736/736 (100%)   \| **rs2498786 allele frequencies (n=736)** \| \| \| \| \| \| \| \| --- \| --- \| --- \| --- \| --- \| --- \| --- \| \|  \| **All subjects** \| \| **status=Ca** \| \| **status=Co** \| \| \| **Allele** \| **Count** \| **Proportion** \| **Count** \| **Proportion** \| **Count** \| **Proportion** \| \| C \| 1159 \| 0.79 \| 917 \| 0.77 \| 242 \| 0.86 \| \| G \| 313 \| 0.21 \| 273 \| 0.23 \| 40 \| 0.14 \|  \| **rs2498786 genotype frequencies (n=736)** \| \| \| \| \| \| \| \| --- \| --- \| --- \| --- \| --- \| --- \| --- \| \|  \| **All subjects** \| \| **status=Ca** \| \| **status=Co** \| \| \| **Genotype** \| **Count** \| **Proportion** \| **Count** \| **Proportion** \| **Count** \| **Proportion** \| \| C/C \| 459 \| 0.62 \| 356 \| 0.6 \| 103 \| 0.73 \| \| C/G \| 241 \| 0.33 \| 205 \| 0.34 \| 36 \| 0.26 \| \| G/G \| 36 \| 0.05 \| 34 \| 0.06 \| 2 \| 0.01 \|  \| **rs2498786 exact test for Hardy-Weinberg equilibrium (n=736)** \| \| \| \| \| \| \| \| --- \| --- \| --- \| --- \| --- \| --- \| --- \| \|  \| **N11** \| **N12** \| **N22** \| **N1** \| **N2** \| **P-value** \| \| **All subjects** \| 459 \| 241 \| 36 \| 1159 \| 313 \| 0.58 \| \| **status=Ca** \| 356 \| 205 \| 34 \| 917 \| 273 \| 0.56 \| \| **status=Co** \| 103 \| 36 \| 2 \| 242 \| 40 \| 0.74 \|  \| **rs2498786 association with response status (n=736, adjusted by sex)** \| \| \| \| \| \| \| \| \| --- \| --- \| --- \| --- \| --- \| --- \| --- \| --- \| \| **Model** \| **Genotype** \| **status=Ca** \| **status=Co** \| **OR (95% CI)** \| **P-value** \| **AIC** \| **BIC** \| \| Codominant \| C/C \| 356 (59.8%) \| 103 (73%) \| 1.00 \| 0.0023 \| 710.4 \| 728.8 \| \| C/G \| 205 (34.5%) \| 36 (25.5%) \| **0.60 (0.39-0.90)** \| \| G/G \| 34 (5.7%) \| 2 (1.4%) \| **0.20 (0.05-0.85)** \| \| Dominant \| C/C \| 356 (59.8%) \| 103 (73%) \| 1.00 \| 0.0023 \| 711.2 \| 725 \| \| C/G-G/G \| 239 (40.2%) \| 38 (26.9%) \| **0.54 (0.36-0.81)** \| \| Recessive \| C/C-C/G \| 561 (94.3%) \| 139 (98.6%) \| 1.00 \| 0.015 \| 714.6 \| 728.4 \| \| G/G \| 34 (5.7%) \| 2 (1.4%) \| **0.24 (0.06-0.99)** \| \| Overdominant \| C/C-G/G \| 390 (65.5%) \| 105 (74.5%) \| 1.00 \| 0.032 \| 715.9 \| 729.7 \| \| C/G \| 205 (34.5%) \| 36 (25.5%) \| **0.64 (0.42-0.97)** \| \| Log-additive \| --- \| --- \| --- \| **0.55 (0.38-0.79)** \| 7e-04 \| 709 \| 722.8 \|  \| **Interaction analysis with covariate sex** \| \| --- \|  \| **rs2498786 and sex cross-classification interaction table (n=736, crude analysis)** \| \| \| \| \| \| \| \| --- \| --- \| --- \| --- \| --- \| --- \| --- \| \|  \| **female** \| \| \| **male** \| \| \| \|  \| **status=Ca** \| **status=Co** \| **OR (95% CI)** \| **status=Ca** \| **status=Co** \| **OR (95% CI)** \| \| **C/C** \| 186 \| 71 \| 1.00 \| 170 \| 32 \| **0.49 (0.31-0.79)** \| \| **C/G** \| 125 \| 19 \| **0.40 (0.23-0.69)** \| 80 \| 17 \| 0.56 (0.31-1.00) \| \| **G/G** \| 19 \| 2 \| 0.28 (0.06-1.21) \| 15 \| 0 \| 0.00 \| \| **Interaction p-value: 0.026** \| \| \| \| \| \| \|  \| **sex within rs2498786 (n=736, crude analysis)** \| \| \| --- \| --- \| \| **C/C** \| \|  \| **status=Ca** \| **status=Co** \| **OR (95% CI)** \| \| --- \| --- \| --- \| --- \| \| **female** \| 186 \| 71 \| 1.00 \| \| **male** \| 170 \| 32 \| **0.49 (0.31-0.79)** \| \| \| **C/G** \| \|  \| **status=Ca** \| **status=Co** \| **OR (95% CI)** \| \| --- \| --- \| --- \| --- \| \| **female** \| 125 \| 19 \| 1.00 \| \| **male** \| 80 \| 17 \| 1.40 (0.69-2.85) \| \| \| **G/G** \| \|  \| **status=Ca** \| **status=Co** \| **OR (95% CI)** \| \| --- \| --- \| --- \| --- \| \| **female** \| 19 \| 2 \| 1.00 \| \| **male** \| 15 \| 0 \| 0.00 \| \| \| **Test for interaction in the trend:**0.098 \| \|  \| **rs2498786 within sex (n=736, crude analysis)** \| \| \| --- \| --- \| \| **female** \| \|  \| **status=Ca** \| **status=Co** \| **OR (95% CI)** \| \| --- \| --- \| --- \| --- \| \| **C/C** \| 186 \| 71 \| 1.00 \| \| **C/G** \| 125 \| 19 \| **0.40 (0.23-0.69)** \| \| **G/G** \| 19 \| 2 \| 0.28 (0.06-1.21) \| \| \| **male** \| \|  \| **status=Ca** \| **status=Co** \| **OR (95% CI)** \| \| --- \| --- \| --- \| --- \| \| **C/C** \| 170 \| 32 \| 1.00 \| \| **C/G** \| 80 \| 17 \| 1.13 (0.59-2.15) \| \| **G/G** \| 15 \| 0 \| 0.00 \| \| \| **Test for interaction in the trend: 0.026** \| \| |  |
| **SNP:**rs2494752 |  |
| **Percentage of typed samples:**736/736 (100%)   \| **rs2494752 allele frequencies (n=736)** \| \| \| \| \| \| \| \| --- \| --- \| --- \| --- \| --- \| --- \| --- \| \|  \| **All subjects** \| \| **status=Ca** \| \| **status=Co** \| \| \| **Allele** \| **Count** \| **Proportion** \| **Count** \| **Proportion** \| **Count** \| **Proportion** \| \| A \| 1026 \| 0.7 \| 802 \| 0.67 \| 224 \| 0.79 \| \| G \| 446 \| 0.3 \| 388 \| 0.33 \| 58 \| 0.21 \|  \| **rs2494752 genotype frequencies (n=736)** \| \| \| \| \| \| \| \| --- \| --- \| --- \| --- \| --- \| --- \| --- \| \|  \| **All subjects** \| \| **status=Ca** \| \| **status=Co** \| \| \| **Genotype** \| **Count** \| **Proportion** \| **Count** \| **Proportion** \| **Count** \| **Proportion** \| \| A/A \| 371 \| 0.5 \| 280 \| 0.47 \| 91 \| 0.65 \| \| A/G \| 284 \| 0.39 \| 242 \| 0.41 \| 42 \| 0.3 \| \| G/G \| 81 \| 0.11 \| 73 \| 0.12 \| 8 \| 0.06 \|  \| **rs2494752 exact test for Hardy-Weinberg equilibrium (n=736)** \| \| \| \| \| \| \| \| --- \| --- \| --- \| --- \| --- \| --- \| --- \| \|  \| **N11** \| **N12** \| **N22** \| **N1** \| **N2** \| **P-value** \| \| **All subjects** \| 371 \| 284 \| 81 \| 1026 \| 446 \| **0.023** \| \| **status=Ca** \| 280 \| 242 \| 73 \| 802 \| 388 \| 0.076 \| \| **status=Co** \| 91 \| 42 \| 8 \| 224 \| 58 \| 0.3 \|  \| **rs2494752 association with response status (n=736, adjusted by sex)** \| \| \| \| \| \| \| \| \| --- \| --- \| --- \| --- \| --- \| --- \| --- \| --- \| \| **Model** \| **Genotype** \| **status=Ca** \| **status=Co** \| **OR (95% CI)** \| **P-value** \| **AIC** \| **BIC** \| \| Codominant \| A/A \| 280 (47.1%) \| 91 (64.5%) \| 1.00 \| 5e-04 \| 707.2 \| 725.6 \| \| A/G \| 242 (40.7%) \| 42 (29.8%) \| **0.53 (0.36-0.80)** \| \| G/G \| 73 (12.3%) \| 8 (5.7%) \| **0.34 (0.16-0.73)** \| \| Dominant \| A/A \| 280 (47.1%) \| 91 (64.5%) \| 1.00 \| 2e-04 \| 706.5 \| 720.3 \| \| A/G-G/G \| 315 (52.9%) \| 50 (35.5%) \| **0.49 (0.33-0.72)** \| \| Recessive \| A/A-A/G \| 522 (87.7%) \| 133 (94.3%) \| 1.00 \| 0.016 \| 714.7 \| 728.5 \| \| G/G \| 73 (12.3%) \| 8 (5.7%) \| **0.43 (0.20-0.91)** \| \| Overdominant \| A/A-G/G \| 353 (59.3%) \| 99 (70.2%) \| 1.00 \| 0.016 \| 714.8 \| 728.6 \| \| A/G \| 242 (40.7%) \| 42 (29.8%) \| **0.62 (0.42-0.92)** \| \| Log-additive \| --- \| --- \| --- \| **0.56 (0.41-0.76)** \| 1e-04 \| 705.3 \| 719.1 \|  \| **Interaction analysis with covariate sex** \| \| --- \|  \| **rs2494752 and sex cross-classification interaction table (n=736, crude analysis)** \| \| \| \| \| \| \| \| --- \| --- \| --- \| --- \| --- \| --- \| --- \| \|  \| **female** \| \| \| **male** \| \| \| \|  \| **status=Ca** \| **status=Co** \| **OR (95% CI)** \| **status=Ca** \| **status=Co** \| **OR (95% CI)** \| \| **A/A** \| 155 \| 60 \| 1.00 \| 125 \| 31 \| 0.64 (0.39-1.05) \| \| **A/G** \| 133 \| 28 \| **0.54 (0.33-0.90)** \| 109 \| 14 \| **0.33 (0.18-0.62)** \| \| **G/G** \| 42 \| 4 \| **0.25 (0.08-0.72)** \| 31 \| 4 \| **0.33 (0.11-0.98)** \| \| **Interaction p-value:**0.61 \| \| \| \| \| \| \|  \| **sex within rs2494752 (n=736, crude analysis)** \| \| \| --- \| --- \| \| **A/A** \| \|  \| **status=Ca** \| **status=Co** \| **OR (95% CI)** \| \| --- \| --- \| --- \| --- \| \| **female** \| 155 \| 60 \| 1.00 \| \| **male** \| 125 \| 31 \| 0.64 (0.39-1.05) \| \| \| **A/G** \| \|  \| **status=Ca** \| **status=Co** \| **OR (95% CI)** \| \| --- \| --- \| --- \| --- \| \| **female** \| 133 \| 28 \| 1.00 \| \| **male** \| 109 \| 14 \| 0.61 (0.31-1.22) \| \| \| **G/G** \| \|  \| **status=Ca** \| **status=Co** \| **OR (95% CI)** \| \| --- \| --- \| --- \| --- \| \| **female** \| 42 \| 4 \| 1.00 \| \| **male** \| 31 \| 4 \| 1.35 (0.31-5.84) \| \| \| **Test for interaction in the trend:**0.56 \| \|  \| **rs2494752 within sex (n=736, crude analysis)** \| \| \| --- \| --- \| \| **female** \| \|  \| **status=Ca** \| **status=Co** \| **OR (95% CI)** \| \| --- \| --- \| --- \| --- \| \| **A/A** \| 155 \| 60 \| 1.00 \| \| **A/G** \| 133 \| 28 \| **0.54 (0.33-0.90)** \| \| **G/G** \| 42 \| 4 \| **0.25 (0.08-0.72)** \| \| \| **male** \| \|  \| **status=Ca** \| **status=Co** \| **OR (95% CI)** \| \| --- \| --- \| --- \| --- \| \| **A/A** \| 125 \| 31 \| 1.00 \| \| **A/G** \| 109 \| 14 \| 0.52 (0.26-1.02) \| \| **G/G** \| 31 \| 4 \| 0.52 (0.17-1.58) \| \| \| **Test for interaction in the trend:**0.61 \| \| |  |
| **SNP:**rs5811155 |  |
| **Percentage of typed samples:**736/736 (100%)   \| **rs5811155 allele frequencies (n=736)** \| \| \| \| \| \| \| \| --- \| --- \| --- \| --- \| --- \| --- \| --- \| \|  \| **All subjects** \| \| **status=Ca** \| \| **status=Co** \| \| \| **Allele** \| **Count** \| **Proportion** \| **Count** \| **Proportion** \| **Count** \| **Proportion** \| \| C \| 994 \| 0.68 \| 773 \| 0.65 \| 221 \| 0.78 \| \| T \| 478 \| 0.32 \| 417 \| 0.35 \| 61 \| 0.22 \|  \| **rs5811155 genotype frequencies (n=736)** \| \| \| \| \| \| \| \| --- \| --- \| --- \| --- \| --- \| --- \| --- \| \|  \| **All subjects** \| \| **status=Ca** \| \| **status=Co** \| \| \| **Genotype** \| **Count** \| **Proportion** \| **Count** \| **Proportion** \| **Count** \| **Proportion** \| \| C/C \| 348 \| 0.47 \| 260 \| 0.44 \| 88 \| 0.62 \| \| C/T \| 298 \| 0.4 \| 253 \| 0.43 \| 45 \| 0.32 \| \| T/T \| 90 \| 0.12 \| 82 \| 0.14 \| 8 \| 0.06 \|  \| **rs5811155 exact test for Hardy-Weinberg equilibrium (n=736)** \| \| \| \| \| \| \| \| --- \| --- \| --- \| --- \| --- \| --- \| --- \| \|  \| **N11** \| **N12** \| **N22** \| **N1** \| **N2** \| **P-value** \| \| **All subjects** \| 348 \| 298 \| 90 \| 994 \| 478 \| **0.043** \| \| **status=Ca** \| 260 \| 253 \| 82 \| 773 \| 417 \| 0.11 \| \| **status=Co** \| 88 \| 45 \| 8 \| 221 \| 61 \| 0.46 \|  \| **rs5811155 association with response status (n=736, adjusted by sex)** \| \| \| \| \| \| \| \| \| --- \| --- \| --- \| --- \| --- \| --- \| --- \| --- \| \| **Model** \| **Genotype** \| **status=Ca** \| **status=Co** \| **OR (95% CI)** \| **P-value** \| **AIC** \| **BIC** \| \| Codominant \| C/C \| 260 (43.7%) \| 88 (62.4%) \| 1.00 \| 1e-04 \| 704.1 \| 722.5 \| \| C/T \| 253 (42.5%) \| 45 (31.9%) \| **0.52 (0.35-0.78)** \| \| T/T \| 82 (13.8%) \| 8 (5.7%) \| **0.29 (0.13-0.62)** \| \| Dominant \| C/C \| 260 (43.7%) \| 88 (62.4%) \| 1.00 \| 1e-04 \| 704.4 \| 718.2 \| \| C/T-T/T \| 335 (56.3%) \| 53 (37.6%) \| **0.47 (0.32-0.68)** \| \| Recessive \| C/C-C/T \| 513 (86.2%) \| 133 (94.3%) \| 1.00 \| 0.0048 \| 712.6 \| 726.4 \| \| T/T \| 82 (13.8%) \| 8 (5.7%) \| **0.38 (0.18-0.80)** \| \| Overdominant \| C/C-T/T \| 342 (57.5%) \| 96 (68.1%) \| 1.00 \| 0.018 \| 714.9 \| 728.7 \| \| C/T \| 253 (42.5%) \| 45 (31.9%) \| **0.63 (0.43-0.93)** \| \| Log-additive \| --- \| --- \| --- \| **0.53 (0.39-0.72)** \| <0.0001 \| 702.1 \| 715.9 \|  \| **Interaction analysis with covariate sex** \| \| --- \|  \| **rs5811155 and sex cross-classification interaction table (n=736, crude analysis)** \| \| \| \| \| \| \| \| --- \| --- \| --- \| --- \| --- \| --- \| --- \| \|  \| **female** \| \| \| **male** \| \| \| \|  \| **status=Ca** \| **status=Co** \| **OR (95% CI)** \| **status=Ca** \| **status=Co** \| **OR (95% CI)** \| \| **C/C** \| 141 \| 59 \| 1.00 \| 119 \| 29 \| **0.58 (0.35-0.97)** \| \| **C/T** \| 144 \| 29 \| **0.48 (0.29-0.79)** \| 109 \| 16 \| **0.35 (0.19-0.64)** \| \| **T/T** \| 45 \| 4 \| **0.21 (0.07-0.62)** \| 37 \| 4 \| **0.26 (0.09-0.76)** \| \| **Interaction p-value:**0.61 \| \| \| \| \| \| \|  \| **sex within rs5811155 (n=736, crude analysis)** \| \| \| --- \| --- \| \| **C/C** \| \|  \| **status=Ca** \| **status=Co** \| **OR (95% CI)** \| \| --- \| --- \| --- \| --- \| \| **female** \| 141 \| 59 \| 1.00 \| \| **male** \| 119 \| 29 \| **0.58 (0.35-0.97)** \| \| \| **C/T** \| \|  \| **status=Ca** \| **status=Co** \| **OR (95% CI)** \| \| --- \| --- \| --- \| --- \| \| **female** \| 144 \| 29 \| 1.00 \| \| **male** \| 109 \| 16 \| 0.73 (0.38-1.41) \| \| \| **T/T** \| \|  \| **status=Ca** \| **status=Co** \| **OR (95% CI)** \| \| --- \| --- \| --- \| --- \| \| **female** \| 45 \| 4 \| 1.00 \| \| **male** \| 37 \| 4 \| 1.22 (0.28-5.20) \| \| \| **Test for interaction in the trend:**0.34 \| \|  \| **rs5811155 within sex (n=736, crude analysis)** \| \| \| --- \| --- \| \| **female** \| \|  \| **status=Ca** \| **status=Co** \| **OR (95% CI)** \| \| --- \| --- \| --- \| --- \| \| **C/C** \| 141 \| 59 \| 1.00 \| \| **C/T** \| 144 \| 29 \| **0.48 (0.29-0.79)** \| \| **T/T** \| 45 \| 4 \| **0.21 (0.07-0.62)** \| \| \| **male** \| \|  \| **status=Ca** \| **status=Co** \| **OR (95% CI)** \| \| --- \| --- \| --- \| --- \| \| **C/C** \| 119 \| 29 \| 1.00 \| \| **C/T** \| 109 \| 16 \| 0.60 (0.31-1.17) \| \| **T/T** \| 37 \| 4 \| 0.44 (0.15-1.34) \| \| \| **Test for interaction in the trend:**0.61 \| \| |  |
| **Multiple-SNP analysis** |  |
| \| **Linkage disequilibrium analysis** \| \| --- \|   **D statistic**   \|  \| rs2498786 \| rs2494752 \| rs5811155 \| \| --- \| --- \| --- \| --- \| \| rs2498786 \| . \| 0.1269 \| 0.1435 \| \| rs2494752 \| . \| . \| 0.2046 \| \| rs5811155 \| . \| . \| . \|   **D' statistic**   \|  \| rs2498786 \| rs2494752 \| rs5811155 \| \| --- \| --- \| --- \| --- \| \| rs2498786 \| . \| 0.8564 \| 0.9996 \| \| rs2494752 \| . \| . \| 0.9998 \| \| rs5811155 \| . \| . \| . \|   **r statistic**   \|  \| rs2498786 \| rs2494752 \| rs5811155 \| \| --- \| --- \| --- \| --- \| \| rs2498786 \| . \| 0.675 \| 0.7491 \| \| rs2494752 \| . \| . \| 0.9506 \| \| rs5811155 \| . \| . \| . \|   **P-values**   \|  \| rs2498786 \| rs2494752 \| rs5811155 \| \| --- \| --- \| --- \| --- \| \| rs2498786 \| . \| 0 \| 0 \| \| rs2494752 \| . \| . \| 0 \| \| rs5811155 \| . \| . \| . \| |  |
| \| **Haplotype analysis** \| \| --- \| |  |
| \| **Haplotype frequencies estimation (n=736)** \| \| \| \| \| \| \| \| \| --- \| --- \| --- \| --- \| --- \| --- \| --- \| --- \| \|  \| **rs2498786** \| **rs2494752** \| **rs5811155** \| **Total** \| **group.Ca** \| **group.Co** \| **Cumulative frequency** \| \| 1 \| C \| A \| C \| 0.6753 \| 0.6496 \| 0.7837 \| 0.6753 \| \| 2 \| G \| G \| T \| 0.1909 \| 0.205 \| 0.1312 \| 0.8662 \| \| 3 \| C \| G \| T \| 0.1121 \| 0.121 \| 0.0745 \| 0.9783 \| \| 4 \| G \| A \| T \| 0.0217 \| 0.0244 \| 0.0106 \| 1 \| \| 5 \| C \| A \| T \| 0 \| 0 \| NA \| 1 \| |  |
| \| **Haplotype association with response (n=736, adjusted by sex)** \| \| \| \| \| \| \| \| \| \| --- \| --- \| --- \| --- \| --- \| --- \| --- \| --- \| --- \| \|  \| **rs2498786** \| **rs2494752** \| **rs5811155** \| **Freq** \| **OR (95% CI)** \| **P-value** \|  \|  \| \| 1 \| C \| A \| C \| 0.6753 \| 1.00 \| --- \|  \|  \| \| 2 \| G \| G \| T \| 0.1909 \| **0.54 (0.37 - 0.78)** \| 0.0012 \|  \|  \| \| 3 \| C \| G \| T \| 0.1121 \| **0.55 (0.34 - 0.89)** \| 0.014 \|  \|  \| \| 4 \| G \| A \| T \| 0.0217 \| 0.36 (0.11 - 1.19) \| 0.094 \|  \|  \| \| **Global haplotype association p-value: 0.00028** \| \| \| \| \| \| \| \| \| |  |
| \| **Haplotype interaction analysis with covariate sex** \| \| --- \| |  |
| \| **Haplotype and sex cross-classification interaction table (n=736, crude analysis)** \| \| \| \| \| --- \| --- \| --- \| --- \| \|  \|  \| **female** \| **male** \| \| **Haplotype** \| **Frequency** \| **OR (95% CI)** \| **OR (95% CI)** \| \| **CAC** \| 0.6753 \| 1.00 \| **0.57 (0.35 - 0.93)** \| \| **CGT** \| 0.1121 \| 0.60 (0.33 - 1.09) \| **0.28 (0.12 - 0.61)** \| \| **GAT** \| 0.0217 \| 0.15 (0.02 - 1.14) \| 0.46 (0.11 - 1.96) \| \| **GGT** \| 0.1909 \| **0.44 (0.27 - 0.72)** \| **0.42 (0.23 - 0.76)** \| \| **Interaction p-value:**0.29 \| \| \| \| |  |
| \| **Haplotypes within sex (n=736, crude analysis)** \| \| \| \| \| --- \| --- \| --- \| --- \| \|  \|  \| **female** \| **male** \| \| **Haplotype** \| **Frequency** \| **OR (95% CI)** \| **OR (95% CI)** \| \| **CAC** \| 0.6753 \| 1.00 \| 1.00 \| \| **CGT** \| 0.1121 \| 0.60 (0.33 - 1.09) \| 0.49 (0.22 - 1.07) \| \| **GAT** \| 0.0217 \| 0.15 (0.02 - 1.14) \| 0.81 (0.19 - 3.41) \| \| **GGT** \| 0.1909 \| **0.44 (0.27 - 0.72)** \| 0.74 (0.40 - 1.34) \| |  |
| \| **sex within haplotypes (n=736, crude analysis)** \| \| \| \| \| --- \| --- \| --- \| --- \| \|  \|  \| **female** \| **male** \| \| **Haplotype** \| **Frequency** \| **OR (95% CI)** \| **OR (95% CI)** \| \| **CAC** \| 0.6753 \| 1.00 \| **0.57 (0.35 - 0.93)** \| \| **CGT** \| 0.1121 \| 1.00 \| 0.46 (0.18 - 1.15) \| \| **GAT** \| 0.0217 \| 1.00 \| 3.08 (0.26 - 36.35) \| \| **GGT** \| 0.1909 \| 1.00 \| 0.94 (0.48 - 1.84) \| |  |

1. Association between AKT1 and the risk of MPA in Guangxi

| SNPStats results | |
| --- | --- |
| **Index** |  |
| \| [**Descriptive statistics**](https://www.snpstats.net/analyzer.php#covdesc) \| \| \| --- \| --- \| \| [**Single-SNP analysis**](https://www.snpstats.net/analyzer.php#singlesnp) \| \| \|  \| [rs2498786](https://www.snpstats.net/analyzer.php#snp1) \| \|  \| [rs2494752](https://www.snpstats.net/analyzer.php#snp2) \| \|  \| [rs5811155](https://www.snpstats.net/analyzer.php#snp3) \| \| [**Multiple-SNP analysis**](https://www.snpstats.net/analyzer.php#multiplesnp) \| \| \|  \| [Linkage disequilibrium analysis](https://www.snpstats.net/analyzer.php#ldanalysis) \| \|  \| [Haplotype analysis](https://www.snpstats.net/analyzer.php#haploanalysis) \| |  |
| **WARNING: 4 observations with missing response value removed from association analyses.** |  |
| **Descriptive statistics** |  |
| **Response variable:**status **Type:**categorical |  |
| \|  \| **n** \| **missing** \| **unique** \| \| --- \| --- \| --- \| --- \| \| **All subjects** \| 420 \| 4 \| 2 \| \| **status=Ca** \| 208 (50%) \| --- \| --- \| \| **status=Co** \| 208 (50%) \| --- \| --- \| |  |
| **Covariate:**sex **Type:**categorical |  |
| \|  \| **n** \| **missing** \| **unique** \| \| --- \| --- \| --- \| --- \| \| **All subjects** \| 420 \| 4 \| 2 \| \| **status=Ca** \| 208 \| 4 \| 2 \| \| **status=Co** \| 208 \| 4 \| 2 \|  \|  \| **female** \| **male** \| \| --- \| --- \| --- \| \| **All subjects** \| 257 (62%) \| 159 (38%) \| \| **status=Ca** \| 127 (61%) \| 81 (39%) \| \| **status=Co** \| 130 (62%) \| 78 (38%) \| |  |
| **Single-SNP analysis** |  |
| **SNP:**rs2498786 |  |
| **Percentage of typed samples:**416/420 (99.05%)   \| **rs2498786 allele frequencies (n=416)** \| \| \| \| \| \| \| \| --- \| --- \| --- \| --- \| --- \| --- \| --- \| \|  \| **All subjects** \| \| **status=Ca** \| \| **status=Co** \| \| \| **Allele** \| **Count** \| **Proportion** \| **Count** \| **Proportion** \| **Count** \| **Proportion** \| \| C \| 674 \| 0.81 \| 319 \| 0.77 \| 355 \| 0.85 \| \| G \| 158 \| 0.19 \| 97 \| 0.23 \| 61 \| 0.15 \|  \| **rs2498786 genotype frequencies (n=420)** \| \| \| \| \| \| \| \| --- \| --- \| --- \| --- \| --- \| --- \| --- \| \|  \| **All subjects** \| \| **status=Ca** \| \| **status=Co** \| \| \| **Genotype** \| **Count** \| **Proportion** \| **Count** \| **Proportion** \| **Count** \| **Proportion** \| \| C/C \| 267 \| 0.64 \| 117 \| 0.56 \| 150 \| 0.72 \| \| C/G \| 140 \| 0.34 \| 85 \| 0.41 \| 55 \| 0.26 \| \| G/G \| 9 \| 0.02 \| 6 \| 0.03 \| 3 \| 0.01 \| \| NA \| 4 \| --- \| 0 \| --- \| 0 \| --- \|  \| **rs2498786 exact test for Hardy-Weinberg equilibrium (n=416)** \| \| \| \| \| \| \| \| --- \| --- \| --- \| --- \| --- \| --- \| --- \| \|  \| **N11** \| **N12** \| **N22** \| **N1** \| **N2** \| **P-value** \| \| **All subjects** \| 267 \| 140 \| 9 \| 674 \| 158 \| 0.078 \| \| **status=Ca** \| 117 \| 85 \| 6 \| 319 \| 97 \| 0.051 \| \| **status=Co** \| 150 \| 55 \| 3 \| 355 \| 61 \| 0.58 \|  \| **rs2498786 association with response status (n=416, adjusted by sex)** \| \| \| \| \| \| \| \| \| --- \| --- \| --- \| --- \| --- \| --- \| --- \| --- \| \| **Model** \| **Genotype** \| **status=Ca** \| **status=Co** \| **OR (95% CI)** \| **P-value** \| **AIC** \| **BIC** \| \| Codominant \| C/C \| 117 (56.2%) \| 150 (72.1%) \| 1.00 \| 0.0032 \| 573.1 \| 589.2 \| \| C/G \| 85 (40.9%) \| 55 (26.4%) \| **0.51 (0.33-0.77)** \| \| G/G \| 6 (2.9%) \| 3 (1.4%) \| 0.39 (0.10-1.60) \| \| Dominant \| C/C \| 117 (56.2%) \| 150 (72.1%) \| 1.00 \| 7e-04 \| 571.2 \| 583.3 \| \| C/G-G/G \| 91 (43.8%) \| 58 (27.9%) \| **0.50 (0.33-0.75)** \| \| Recessive \| C/C-C/G \| 202 (97.1%) \| 205 (98.6%) \| 1.00 \| 0.31 \| 581.6 \| 593.7 \| \| G/G \| 6 (2.9%) \| 3 (1.4%) \| 0.50 (0.12-2.02) \| \| Overdominant \| C/C-G/G \| 123 (59.1%) \| 153 (73.6%) \| 1.00 \| 0.0019 \| 572.9 \| 585 \| \| C/G \| 85 (40.9%) \| 55 (26.4%) \| **0.52 (0.34-0.79)** \| \| Log-additive \| --- \| --- \| --- \| **0.53 (0.36-0.77)** \| 8e-04 \| 571.4 \| 583.5 \|  \| **Interaction analysis with covariate sex** \| \| --- \|  \| **rs2498786 and sex cross-classification interaction table (n=416, crude analysis)** \| \| \| \| \| \| \| \| --- \| --- \| --- \| --- \| --- \| --- \| --- \| \|  \| **female** \| \| \| **male** \| \| \| \|  \| **status=Ca** \| **status=Co** \| **OR (95% CI)** \| **status=Ca** \| **status=Co** \| **OR (95% CI)** \| \| **C/C** \| 69 \| 101 \| 1.00 \| 48 \| 49 \| 0.70 (0.42-1.15) \| \| **C/G** \| 56 \| 27 \| **0.33 (0.19-0.57)** \| 29 \| 28 \| 0.66 (0.36-1.21) \| \| **G/G** \| 2 \| 2 \| 0.68 (0.09-4.97) \| 4 \| 1 \| 0.17 (0.02-1.56) \| \| **Interaction p-value: 0.034** \| \| \| \| \| \| \|  \| **sex within rs2498786 (n=416, crude analysis)** \| \| \| --- \| --- \| \| **C/C** \| \|  \| **status=Ca** \| **status=Co** \| **OR (95% CI)** \| \| --- \| --- \| --- \| --- \| \| **female** \| 69 \| 101 \| 1.00 \| \| **male** \| 48 \| 49 \| 0.70 (0.42-1.15) \| \| \| **C/G** \| \|  \| **status=Ca** \| **status=Co** \| **OR (95% CI)** \| \| --- \| --- \| --- \| --- \| \| **female** \| 56 \| 27 \| 1.00 \| \| **male** \| 29 \| 28 \| 2.00 (1.00-4.01) \| \| \| **G/G** \| \|  \| **status=Ca** \| **status=Co** \| **OR (95% CI)** \| \| --- \| --- \| --- \| --- \| \| **female** \| 2 \| 2 \| 1.00 \| \| **male** \| 4 \| 1 \| 0.25 (0.01-4.73) \| \| \| **Test for interaction in the trend:**0.07 \| \|  \| **rs2498786 within sex (n=416, crude analysis)** \| \| \| --- \| --- \| \| **female** \| \|  \| **status=Ca** \| **status=Co** \| **OR (95% CI)** \| \| --- \| --- \| --- \| --- \| \| **C/C** \| 69 \| 101 \| 1.00 \| \| **C/G** \| 56 \| 27 \| **0.33 (0.19-0.57)** \| \| **G/G** \| 2 \| 2 \| 0.68 (0.09-4.97) \| \| \| **male** \| \|  \| **status=Ca** \| **status=Co** \| **OR (95% CI)** \| \| --- \| --- \| --- \| --- \| \| **C/C** \| 48 \| 49 \| 1.00 \| \| **C/G** \| 29 \| 28 \| 0.95 (0.49-1.82) \| \| **G/G** \| 4 \| 1 \| 0.24 (0.03-2.27) \| \| \| **Test for interaction in the trend: 0.034** \| \| |  |
| **SNP:**rs2494752 |  |
| **Percentage of typed samples:**416/420 (99.05%)   \| **rs2494752 allele frequencies (n=416)** \| \| \| \| \| \| \| \| --- \| --- \| --- \| --- \| --- \| --- \| --- \| \|  \| **All subjects** \| \| **status=Ca** \| \| **status=Co** \| \| \| **Allele** \| **Count** \| **Proportion** \| **Count** \| **Proportion** \| **Count** \| **Proportion** \| \| A \| 616 \| 0.74 \| 291 \| 0.7 \| 325 \| 0.78 \| \| G \| 216 \| 0.26 \| 125 \| 0.3 \| 91 \| 0.22 \|  \| **rs2494752 genotype frequencies (n=420)** \| \| \| \| \| \| \| \| --- \| --- \| --- \| --- \| --- \| --- \| --- \| \|  \| **All subjects** \| \| **status=Ca** \| \| **status=Co** \| \| \| **Genotype** \| **Count** \| **Proportion** \| **Count** \| **Proportion** \| **Count** \| **Proportion** \| \| A/A \| 230 \| 0.55 \| 101 \| 0.49 \| 129 \| 0.62 \| \| A/G \| 156 \| 0.38 \| 89 \| 0.43 \| 67 \| 0.32 \| \| G/G \| 30 \| 0.07 \| 18 \| 0.09 \| 12 \| 0.06 \| \| NA \| 4 \| --- \| 0 \| --- \| 0 \| --- \|  \| **rs2494752 exact test for Hardy-Weinberg equilibrium (n=416)** \| \| \| \| \| \| \| \| --- \| --- \| --- \| --- \| --- \| --- \| --- \| \|  \| **N11** \| **N12** \| **N22** \| **N1** \| **N2** \| **P-value** \| \| **All subjects** \| 230 \| 156 \| 30 \| 616 \| 216 \| 0.61 \| \| **status=Ca** \| 101 \| 89 \| 18 \| 291 \| 125 \| 0.87 \| \| **status=Co** \| 129 \| 67 \| 12 \| 325 \| 91 \| 0.42 \|  \| **rs2494752 association with response status (n=416, adjusted by sex)** \| \| \| \| \| \| \| \| \| --- \| --- \| --- \| --- \| --- \| --- \| --- \| --- \| \| **Model** \| **Genotype** \| **status=Ca** \| **status=Co** \| **OR (95% CI)** \| **P-value** \| **AIC** \| **BIC** \| \| Codominant \| A/A \| 101 (48.6%) \| 129 (62%) \| 1.00 \| 0.021 \| 576.9 \| 593 \| \| A/G \| 89 (42.8%) \| 67 (32.2%) \| **0.59 (0.39-0.89)** \| \| G/G \| 18 (8.7%) \| 12 (5.8%) \| 0.52 (0.24-1.14) \| \| Dominant \| A/A \| 101 (48.6%) \| 129 (62%) \| 1.00 \| 0.0059 \| 575 \| 587.1 \| \| A/G-G/G \| 107 (51.4%) \| 79 (38%) \| **0.58 (0.39-0.86)** \| \| Recessive \| A/A-A/G \| 190 (91.3%) \| 196 (94.2%) \| 1.00 \| 0.26 \| 581.3 \| 593.4 \| \| G/G \| 18 (8.7%) \| 12 (5.8%) \| 0.65 (0.30-1.38) \| \| Overdominant \| A/A-G/G \| 119 (57.2%) \| 141 (67.8%) \| 1.00 \| 0.026 \| 577.7 \| 589.8 \| \| A/G \| 89 (42.8%) \| 67 (32.2%) \| **0.64 (0.43-0.95)** \| \| Log-additive \| --- \| --- \| --- \| **0.66 (0.48-0.90)** \| 0.0079 \| 575.5 \| 587.6 \|  \| **Interaction analysis with covariate sex** \| \| --- \|  \| **rs2494752 and sex cross-classification interaction table (n=416, crude analysis)** \| \| \| \| \| \| \| \| --- \| --- \| --- \| --- \| --- \| --- \| --- \| \|  \| **female** \| \| \| **male** \| \| \| \|  \| **status=Ca** \| **status=Co** \| **OR (95% CI)** \| **status=Ca** \| **status=Co** \| **OR (95% CI)** \| \| **A/A** \| 61 \| 85 \| 1.00 \| 40 \| 44 \| 0.79 (0.46-1.35) \| \| **A/G** \| 53 \| 40 \| **0.54 (0.32-0.92)** \| 36 \| 27 \| **0.54 (0.30-0.98)** \| \| **G/G** \| 13 \| 5 \| **0.28 (0.09-0.81)** \| 5 \| 7 \| 1.00 (0.30-3.32) \| \| **Interaction p-value:**0.17 \| \| \| \| \| \| \|  \| **sex within rs2494752 (n=416, crude analysis)** \| \| \| --- \| --- \| \| **A/A** \| \|  \| **status=Ca** \| **status=Co** \| **OR (95% CI)** \| \| --- \| --- \| --- \| --- \| \| **female** \| 61 \| 85 \| 1.00 \| \| **male** \| 40 \| 44 \| 0.79 (0.46-1.35) \| \| \| **A/G** \| \|  \| **status=Ca** \| **status=Co** \| **OR (95% CI)** \| \| --- \| --- \| --- \| --- \| \| **female** \| 53 \| 40 \| 1.00 \| \| **male** \| 36 \| 27 \| 0.99 (0.52-1.90) \| \| \| **G/G** \| \|  \| **status=Ca** \| **status=Co** \| **OR (95% CI)** \| \| --- \| --- \| --- \| --- \| \| **female** \| 13 \| 5 \| 1.00 \| \| **male** \| 5 \| 7 \| 3.64 (0.78-17.03) \| \| \| **Test for interaction in the trend:**0.11 \| \|  \| **rs2494752 within sex (n=416, crude analysis)** \| \| \| --- \| --- \| \| **female** \| \|  \| **status=Ca** \| **status=Co** \| **OR (95% CI)** \| \| --- \| --- \| --- \| --- \| \| **A/A** \| 61 \| 85 \| 1.00 \| \| **A/G** \| 53 \| 40 \| **0.54 (0.32-0.92)** \| \| **G/G** \| 13 \| 5 \| **0.28 (0.09-0.81)** \| \| \| **male** \| \|  \| **status=Ca** \| **status=Co** \| **OR (95% CI)** \| \| --- \| --- \| --- \| --- \| \| **A/A** \| 40 \| 44 \| 1.00 \| \| **A/G** \| 36 \| 27 \| 0.68 (0.35-1.32) \| \| **G/G** \| 5 \| 7 \| 1.27 (0.37-4.33) \| \| \| **Test for interaction in the trend:**0.17 \| \| |  |
| **SNP:**rs5811155 |  |
| **Percentage of typed samples:**416/420 (99.05%)   \| **rs5811155 allele frequencies (n=416)** \| \| \| \| \| \| \| \| --- \| --- \| --- \| --- \| --- \| --- \| --- \| \|  \| **All subjects** \| \| **status=Ca** \| \| **status=Co** \| \| \| **Allele** \| **Count** \| **Proportion** \| **Count** \| **Proportion** \| **Count** \| **Proportion** \| \| C \| 606 \| 0.73 \| 284 \| 0.68 \| 322 \| 0.77 \| \| T \| 226 \| 0.27 \| 132 \| 0.32 \| 94 \| 0.23 \|  \| **rs5811155 genotype frequencies (n=420)** \| \| \| \| \| \| \| \| --- \| --- \| --- \| --- \| --- \| --- \| --- \| \|  \| **All subjects** \| \| **status=Ca** \| \| **status=Co** \| \| \| **Genotype** \| **Count** \| **Proportion** \| **Count** \| **Proportion** \| **Count** \| **Proportion** \| \| C/C \| 222 \| 0.53 \| 96 \| 0.46 \| 126 \| 0.61 \| \| C/T \| 162 \| 0.39 \| 92 \| 0.44 \| 70 \| 0.34 \| \| T/T \| 32 \| 0.08 \| 20 \| 0.1 \| 12 \| 0.06 \| \| NA \| 4 \| --- \| 0 \| --- \| 0 \| --- \|  \| **rs5811155 exact test for Hardy-Weinberg equilibrium (n=416)** \| \| \| \| \| \| \| \| --- \| --- \| --- \| --- \| --- \| --- \| --- \| \|  \| **N11** \| **N12** \| **N22** \| **N1** \| **N2** \| **P-value** \| \| **All subjects** \| 222 \| 162 \| 32 \| 606 \| 226 \| 0.71 \| \| **status=Ca** \| 96 \| 92 \| 20 \| 284 \| 132 \| 0.87 \| \| **status=Co** \| 126 \| 70 \| 12 \| 322 \| 94 \| 0.56 \|  \| **rs5811155 association with response status (n=416, adjusted by sex)** \| \| \| \| \| \| \| \| \| --- \| --- \| --- \| --- \| --- \| --- \| --- \| --- \| \| **Model** \| **Genotype** \| **status=Ca** \| **status=Co** \| **OR (95% CI)** \| **P-value** \| **AIC** \| **BIC** \| \| Codominant \| C/C \| 96 (46.1%) \| 126 (60.6%) \| 1.00 \| 0.011 \| 575.6 \| 591.7 \| \| C/T \| 92 (44.2%) \| 70 (33.6%) \| **0.58 (0.39-0.87)** \| \| T/T \| 20 (9.6%) \| 12 (5.8%) \| **0.46 (0.21-0.98)** \| \| Dominant \| C/C \| 96 (46.1%) \| 126 (60.6%) \| 1.00 \| 0.0032 \| 573.9 \| 586 \| \| C/T-T/T \| 112 (53.9%) \| 82 (39.4%) \| **0.56 (0.38-0.82)** \| \| Recessive \| C/C-C/T \| 188 (90.4%) \| 196 (94.2%) \| 1.00 \| 0.14 \| 580.4 \| 592.5 \| \| T/T \| 20 (9.6%) \| 12 (5.8%) \| 0.58 (0.27-1.21) \| \| Overdominant \| C/C-T/T \| 116 (55.8%) \| 138 (66.3%) \| 1.00 \| 0.027 \| 577.7 \| 589.8 \| \| C/T \| 92 (44.2%) \| 70 (33.6%) \| **0.64 (0.43-0.95)** \| \| Log-additive \| --- \| --- \| --- \| **0.63 (0.46-0.86)** \| 0.0033 \| 574 \| 586 \|  \| **Interaction analysis with covariate sex** \| \| --- \|  \| **rs5811155 and sex cross-classification interaction table (n=416, crude analysis)** \| \| \| \| \| \| \| \| --- \| --- \| --- \| --- \| --- \| --- \| --- \| \|  \| **female** \| \| \| **male** \| \| \| \|  \| **status=Ca** \| **status=Co** \| **OR (95% CI)** \| **status=Ca** \| **status=Co** \| **OR (95% CI)** \| \| **C/C** \| 57 \| 84 \| 1.00 \| 39 \| 42 \| 0.73 (0.42-1.27) \| \| **C/T** \| 57 \| 41 \| **0.49 (0.29-0.82)** \| 35 \| 29 \| 0.56 (0.31-1.02) \| \| **T/T** \| 13 \| 5 \| **0.26 (0.09-0.77)** \| 7 \| 7 \| 0.68 (0.23-2.04) \| \| **Interaction p-value:**0.22 \| \| \| \| \| \| \|  \| **sex within rs5811155 (n=416, crude analysis)** \| \| \| --- \| --- \| \| **C/C** \| \|  \| **status=Ca** \| **status=Co** \| **OR (95% CI)** \| \| --- \| --- \| --- \| --- \| \| **female** \| 57 \| 84 \| 1.00 \| \| **male** \| 39 \| 42 \| 0.73 (0.42-1.27) \| \| \| **C/T** \| \|  \| **status=Ca** \| **status=Co** \| **OR (95% CI)** \| \| --- \| --- \| --- \| --- \| \| **female** \| 57 \| 41 \| 1.00 \| \| **male** \| 35 \| 29 \| 1.15 (0.61-2.17) \| \| \| **T/T** \| \|  \| **status=Ca** \| **status=Co** \| **OR (95% CI)** \| \| --- \| --- \| --- \| --- \| \| **female** \| 13 \| 5 \| 1.00 \| \| **male** \| 7 \| 7 \| 2.60 (0.60-11.31) \| \| \| **Test for interaction in the trend:**0.079 \| \|  \| **rs5811155 within sex (n=416, crude analysis)** \| \| \| --- \| --- \| \| **female** \| \|  \| **status=Ca** \| **status=Co** \| **OR (95% CI)** \| \| --- \| --- \| --- \| --- \| \| **C/C** \| 57 \| 84 \| 1.00 \| \| **C/T** \| 57 \| 41 \| **0.49 (0.29-0.82)** \| \| **T/T** \| 13 \| 5 \| **0.26 (0.09-0.77)** \| \| \| **male** \| \|  \| **status=Ca** \| **status=Co** \| **OR (95% CI)** \| \| --- \| --- \| --- \| --- \| \| **C/C** \| 39 \| 42 \| 1.00 \| \| **C/T** \| 35 \| 29 \| 0.77 (0.40-1.48) \| \| **T/T** \| 7 \| 7 \| 0.93 (0.30-2.89) \| \| \| **Test for interaction in the trend:**0.22 \| \| |  |
| **Multiple-SNP analysis** |  |
| \| **Linkage disequilibrium analysis** \| \| --- \|   **D statistic**   \|  \| rs2498786 \| rs2494752 \| rs5811155 \| \| --- \| --- \| --- \| --- \| \| rs2498786 \| . \| 0.1288 \| 0.1383 \| \| rs2494752 \| . \| . \| 0.189 \| \| rs5811155 \| . \| . \| . \|   **D' statistic**   \|  \| rs2498786 \| rs2494752 \| rs5811155 \| \| --- \| --- \| --- \| --- \| \| rs2498786 \| . \| 0.9157 \| 0.9997 \| \| rs2494752 \| . \| . \| 0.9996 \| \| rs5811155 \| . \| . \| . \|   **r statistic**   \|  \| rs2498786 \| rs2494752 \| rs5811155 \| \| --- \| --- \| --- \| --- \| \| rs2498786 \| . \| 0.7487 \| 0.7926 \| \| rs2494752 \| . \| . \| 0.9693 \| \| rs5811155 \| . \| . \| . \|   **P-values**   \|  \| rs2498786 \| rs2494752 \| rs5811155 \| \| --- \| --- \| --- \| --- \| \| rs2498786 \| . \| 0 \| 0 \| \| rs2494752 \| . \| . \| 0 \| \| rs5811155 \| . \| . \| . \| |  |
| \| **Haplotype analysis** \| \| --- \| |  |
| \| **Haplotype frequencies estimation (n=416)** \| \| \| \| \| \| \| \| \| --- \| --- \| --- \| --- \| --- \| --- \| --- \| --- \| \|  \| **rs2498786** \| **rs2494752** \| **rs5811155** \| **Total** \| **group.Ca** \| **group.Co** \| **Cumulative frequency** \| \| 1 \| C \| A \| C \| 0.7284 \| 0.6827 \| 0.774 \| 0.7284 \| \| 2 \| G \| G \| T \| 0.1779 \| 0.2163 \| 0.1394 \| 0.9063 \| \| 3 \| C \| G \| T \| 0.0817 \| 0.0841 \| 0.0793 \| 0.988 \| \| 4 \| G \| A \| T \| 0.012 \| 0.0168 \| 0.0072 \| 1 \| \| 5 \| C \| A \| T \| 0 \| 0 \| NA \| 1 \| |  |
| \| **Haplotype association with response (n=416, adjusted by sex)** \| \| \| \| \| \| \| \| \| \| --- \| --- \| --- \| --- \| --- \| --- \| --- \| --- \| --- \| \|  \| **rs2498786** \| **rs2494752** \| **rs5811155** \| **Freq** \| **OR (95% CI)** \| **P-value** \|  \|  \| \| 1 \| C \| A \| C \| 0.7284 \| 1.00 \| --- \|  \|  \| \| 2 \| G \| G \| T \| 0.1779 \| **0.54 (0.36 - 0.79)** \| 0.0017 \|  \|  \| \| 3 \| C \| G \| T \| 0.0817 \| 0.86 (0.54 - 1.37) \| 0.52 \|  \|  \| \| 4 \| G \| A \| T \| 0.012 \| 0.35 (0.09 - 1.39) \| 0.14 \|  \|  \| \| **Global haplotype association p-value: 0.0074** \| \| \| \| \| \| \| \| \| |  |
| \| **Haplotype interaction analysis with covariate sex** \| \| --- \| |  |
| \| **Haplotype and sex cross-classification interaction table (n=416, crude analysis)** \| \| \| \| \| --- \| --- \| --- \| --- \| \|  \|  \| **female** \| **male** \| \| **Haplotype** \| **Frequency** \| **OR (95% CI)** \| **OR (95% CI)** \| \| **CAC** \| 0.7284 \| 1.00 \| 0.69 (0.41 - 1.18) \| \| **CGT** \| 0.0817 \| 0.74 (0.41 - 1.35) \| 0.75 (0.34 - 1.66) \| \| **GAT** \| 0.012 \| 0.17 (0.02 - 1.52) \| 0.45 (0.07 - 2.84) \| \| **GGT** \| 0.1779 \| **0.39 (0.23 - 0.66)** \| 0.56 (0.31 - 1.00) \| \| **Interaction p-value:**0.24 \| \| \| \| |  |
| \| **Haplotypes within sex (n=416, crude analysis)** \| \| \| \| \| --- \| --- \| --- \| --- \| \|  \|  \| **female** \| **male** \| \| **Haplotype** \| **Frequency** \| **OR (95% CI)** \| **OR (95% CI)** \| \| **CAC** \| 0.7284 \| 1.00 \| 1.00 \| \| **CGT** \| 0.0817 \| 0.74 (0.41 - 1.35) \| 1.08 (0.50 - 2.31) \| \| **GAT** \| 0.012 \| 0.17 (0.02 - 1.52) \| 0.65 (0.11 - 4.05) \| \| **GGT** \| 0.1779 \| **0.39 (0.23 - 0.66)** \| 0.81 (0.45 - 1.46) \| |  |
| \| **sex within haplotypes (n=416, crude analysis)** \| \| \| \| \| --- \| --- \| --- \| --- \| \|  \|  \| **female** \| **male** \| \| **Haplotype** \| **Frequency** \| **OR (95% CI)** \| **OR (95% CI)** \| \| **CAC** \| 0.7284 \| 1.00 \| 0.69 (0.41 - 1.18) \| \| **CGT** \| 0.0817 \| 1.00 \| 1.01 (0.40 - 2.52) \| \| **GAT** \| 0.012 \| 1.00 \| 2.74 (0.16 - 46.76) \| \| **GGT** \| 0.1779 \| 1.00 \| 1.42 (0.74 - 2.73) \| |  |

1. Association between AKT1 and the risk of MPA in Guangxi (adjusted for age)

| SNPStats results | |
| --- | --- |
| **Index** |  |
| \| [**Descriptive statistics**](https://www.snpstats.net/analyzer.php#covdesc) \| \| \| --- \| --- \| \| [**Single-SNP analysis**](https://www.snpstats.net/analyzer.php#singlesnp) \| \| \|  \| [rs2498786](https://www.snpstats.net/analyzer.php#snp1) \| \|  \| [rs2494752](https://www.snpstats.net/analyzer.php#snp2) \| \|  \| [rs5811155](https://www.snpstats.net/analyzer.php#snp3) \| \| [**Multiple-SNP analysis**](https://www.snpstats.net/analyzer.php#multiplesnp) \| \| \|  \| [Linkage disequilibrium analysis](https://www.snpstats.net/analyzer.php#ldanalysis) \| \|  \| [Haplotype analysis](https://www.snpstats.net/analyzer.php#haploanalysis) \| |  |
| **WARNING: 4 observations with missing response value removed from association analyses.** |  |
| **Descriptive statistics** |  |
| **Response variable:**status **Type:**categorical |  |
| \|  \| **n** \| **missing** \| **unique** \| \| --- \| --- \| --- \| --- \| \| **All subjects** \| 420 \| 4 \| 2 \| \| **status=Ca** \| 208 (50%) \| --- \| --- \| \| **status=Co** \| 208 (50%) \| --- \| --- \| |  |
| **Covariate:**sex **Type:**categorical |  |
| \|  \| **n** \| **missing** \| **unique** \| \| --- \| --- \| --- \| --- \| \| **All subjects** \| 420 \| 4 \| 2 \| \| **status=Ca** \| 208 \| 4 \| 2 \| \| **status=Co** \| 208 \| 4 \| 2 \|  \|  \| **female** \| **male** \| \| --- \| --- \| --- \| \| **All subjects** \| 257 (62%) \| 159 (38%) \| \| **status=Ca** \| 127 (61%) \| 81 (39%) \| \| **status=Co** \| 130 (62%) \| 78 (38%) \| |  |
| **Covariate:**age **Type:**quantitative |  |
| \|  \| **n** \| **missing** \| **unique** \| **mean** \| **.05** \| **.10** \| **.25** \| **.50** \| **.75** \| **.90** \| **.95** \| \| --- \| --- \| --- \| --- \| --- \| --- \| --- \| --- \| --- \| --- \| --- \| --- \| \| **All subjects** \| 420 \| 4 \| 63 \| 52.84 \| 26.75 \| 33 \| 44.75 \| 53.5 \| 63 \| 70 \| 74 \| \| **status = Ca** \| 208 \| 0 \| 57 \| 51.06 \| 30.35 \| 35 \| 44 \| 51 \| 58.25 \| 68 \| 71.65 \| \| **status = Co** \| 208 \| 0 \| 57 \| 54.61 \| 24.05 \| 30 \| 45.75 \| 58 \| 65 \| 72 \| 74.65 \|   lowest: 18, 19, 20, 21, 22 highest: 78, 78, 79, 81, 82 |  |
| **Single-SNP analysis** |  |
| **SNP:**rs2498786 |  |
| **Percentage of typed samples:**416/420 (99.05%)   \| **rs2498786 allele frequencies (n=416)** \| \| \| \| \| \| \| \| --- \| --- \| --- \| --- \| --- \| --- \| --- \| \|  \| **All subjects** \| \| **status=Ca** \| \| **status=Co** \| \| \| **Allele** \| **Count** \| **Proportion** \| **Count** \| **Proportion** \| **Count** \| **Proportion** \| \| C \| 674 \| 0.81 \| 319 \| 0.77 \| 355 \| 0.85 \| \| G \| 158 \| 0.19 \| 97 \| 0.23 \| 61 \| 0.15 \|  \| **rs2498786 genotype frequencies (n=420)** \| \| \| \| \| \| \| \| --- \| --- \| --- \| --- \| --- \| --- \| --- \| \|  \| **All subjects** \| \| **status=Ca** \| \| **status=Co** \| \| \| **Genotype** \| **Count** \| **Proportion** \| **Count** \| **Proportion** \| **Count** \| **Proportion** \| \| C/C \| 267 \| 0.64 \| 117 \| 0.56 \| 150 \| 0.72 \| \| C/G \| 140 \| 0.34 \| 85 \| 0.41 \| 55 \| 0.26 \| \| G/G \| 9 \| 0.02 \| 6 \| 0.03 \| 3 \| 0.01 \| \| NA \| 4 \| --- \| 0 \| --- \| 0 \| --- \|  \| **rs2498786 exact test for Hardy-Weinberg equilibrium (n=416)** \| \| \| \| \| \| \| \| --- \| --- \| --- \| --- \| --- \| --- \| --- \| \|  \| **N11** \| **N12** \| **N22** \| **N1** \| **N2** \| **P-value** \| \| **All subjects** \| 267 \| 140 \| 9 \| 674 \| 158 \| 0.078 \| \| **status=Ca** \| 117 \| 85 \| 6 \| 319 \| 97 \| 0.051 \| \| **status=Co** \| 150 \| 55 \| 3 \| 355 \| 61 \| 0.58 \|  \| **rs2498786 association with response status (n=416, adjusted by sex+age)** \| \| \| \| \| \| \| \| \| --- \| --- \| --- \| --- \| --- \| --- \| --- \| --- \| \| **Model** \| **Genotype** \| **status=Ca** \| **status=Co** \| **OR (95% CI)** \| **P-value** \| **AIC** \| **BIC** \| \| Codominant \| C/C \| 117 (56.2%) \| 150 (72.1%) \| 1.00 \| 0.0082 \| 570 \| 590.2 \| \| C/G \| 85 (40.9%) \| 55 (26.4%) \| **0.53 (0.35-0.81)** \| \| G/G \| 6 (2.9%) \| 3 (1.4%) \| 0.41 (0.10-1.69) \| \| Dominant \| C/C \| 117 (56.2%) \| 150 (72.1%) \| 1.00 \| 0.0021 \| 568.2 \| 584.3 \| \| C/G-G/G \| 91 (43.8%) \| 58 (27.9%) \| **0.52 (0.35-0.79)** \| \| Recessive \| C/C-C/G \| 202 (97.1%) \| 205 (98.6%) \| 1.00 \| 0.34 \| 576.7 \| 592.8 \| \| G/G \| 6 (2.9%) \| 3 (1.4%) \| 0.51 (0.12-2.09) \| \| Overdominant \| C/C-G/G \| 123 (59.1%) \| 153 (73.6%) \| 1.00 \| 0.0048 \| 569.7 \| 585.8 \| \| C/G \| 85 (40.9%) \| 55 (26.4%) \| **0.55 (0.36-0.83)** \| \| Log-additive \| --- \| --- \| --- \| **0.56 (0.38-0.81)** \| 0.0022 \| 568.2 \| 584.4 \|  \| **Interaction analysis with covariate sex** \| \| --- \|  \| **rs2498786 and sex cross-classification interaction table (n=416, adjusted by age)** \| \| \| \| \| \| \| \| --- \| --- \| --- \| --- \| --- \| --- \| --- \| \|  \| **female** \| \| \| **male** \| \| \| \|  \| **status=Ca** \| **status=Co** \| **OR (95% CI)** \| **status=Ca** \| **status=Co** \| **OR (95% CI)** \| \| **C/C** \| 69 \| 101 \| 1.00 \| 48 \| 49 \| 0.67 (0.41-1.12) \| \| **C/G** \| 56 \| 27 \| **0.35 (0.20-0.60)** \| 29 \| 28 \| 0.68 (0.37-1.24) \| \| **G/G** \| 2 \| 2 \| 0.66 (0.09-4.79) \| 4 \| 1 \| 0.18 (0.02-1.69) \| \| **Interaction p-value: 0.037** \| \| \| \| \| \| \|  \| **sex within rs2498786 (n=416, adjusted by age)** \| \| \| --- \| --- \| \| **C/C** \| \|  \| **status=Ca** \| **status=Co** \| **OR (95% CI)** \| \| --- \| --- \| --- \| --- \| \| **female** \| 69 \| 101 \| 1.00 \| \| **male** \| 48 \| 49 \| 0.67 (0.41-1.12) \| \| \| **C/G** \| \|  \| **status=Ca** \| **status=Co** \| **OR (95% CI)** \| \| --- \| --- \| --- \| --- \| \| **female** \| 56 \| 27 \| 1.00 \| \| **male** \| 29 \| 28 \| 1.95 (0.97-3.92) \| \| \| **G/G** \| \|  \| **status=Ca** \| **status=Co** \| **OR (95% CI)** \| \| --- \| --- \| --- \| --- \| \| **female** \| 2 \| 2 \| 1.00 \| \| **male** \| 4 \| 1 \| 0.27 (0.01-5.29) \| \| \| **Test for interaction in the trend:**0.066 \| \|  \| **rs2498786 within sex (n=416, adjusted by age)** \| \| \| --- \| --- \| \| **female** \| \|  \| **status=Ca** \| **status=Co** \| **OR (95% CI)** \| \| --- \| --- \| --- \| --- \| \| **C/C** \| 69 \| 101 \| 1.00 \| \| **C/G** \| 56 \| 27 \| **0.35 (0.20-0.60)** \| \| **G/G** \| 2 \| 2 \| 0.66 (0.09-4.79) \| \| \| **male** \| \|  \| **status=Ca** \| **status=Co** \| **OR (95% CI)** \| \| --- \| --- \| --- \| --- \| \| **C/C** \| 48 \| 49 \| 1.00 \| \| **C/G** \| 29 \| 28 \| 1.00 (0.52-1.94) \| \| **G/G** \| 4 \| 1 \| 0.27 (0.03-2.54) \| \| \| **Test for interaction in the trend: 0.037** \| \| |  |
| **SNP:**rs2494752 |  |
| **Percentage of typed samples:**416/420 (99.05%)   \| **rs2494752 allele frequencies (n=416)** \| \| \| \| \| \| \| \| --- \| --- \| --- \| --- \| --- \| --- \| --- \| \|  \| **All subjects** \| \| **status=Ca** \| \| **status=Co** \| \| \| **Allele** \| **Count** \| **Proportion** \| **Count** \| **Proportion** \| **Count** \| **Proportion** \| \| A \| 616 \| 0.74 \| 291 \| 0.7 \| 325 \| 0.78 \| \| G \| 216 \| 0.26 \| 125 \| 0.3 \| 91 \| 0.22 \|  \| **rs2494752 genotype frequencies (n=420)** \| \| \| \| \| \| \| \| --- \| --- \| --- \| --- \| --- \| --- \| --- \| \|  \| **All subjects** \| \| **status=Ca** \| \| **status=Co** \| \| \| **Genotype** \| **Count** \| **Proportion** \| **Count** \| **Proportion** \| **Count** \| **Proportion** \| \| A/A \| 230 \| 0.55 \| 101 \| 0.49 \| 129 \| 0.62 \| \| A/G \| 156 \| 0.38 \| 89 \| 0.43 \| 67 \| 0.32 \| \| G/G \| 30 \| 0.07 \| 18 \| 0.09 \| 12 \| 0.06 \| \| NA \| 4 \| --- \| 0 \| --- \| 0 \| --- \|  \| **rs2494752 exact test for Hardy-Weinberg equilibrium (n=416)** \| \| \| \| \| \| \| \| --- \| --- \| --- \| --- \| --- \| --- \| --- \| \|  \| **N11** \| **N12** \| **N22** \| **N1** \| **N2** \| **P-value** \| \| **All subjects** \| 230 \| 156 \| 30 \| 616 \| 216 \| 0.61 \| \| **status=Ca** \| 101 \| 89 \| 18 \| 291 \| 125 \| 0.87 \| \| **status=Co** \| 129 \| 67 \| 12 \| 325 \| 91 \| 0.42 \|  \| **rs2494752 association with response status (n=416, adjusted by sex+age)** \| \| \| \| \| \| \| \| \| --- \| --- \| --- \| --- \| --- \| --- \| --- \| --- \| \| **Model** \| **Genotype** \| **status=Ca** \| **status=Co** \| **OR (95% CI)** \| **P-value** \| **AIC** \| **BIC** \| \| Codominant \| A/A \| 101 (48.6%) \| 129 (62%) \| 1.00 \| 0.041 \| 573.2 \| 593.4 \| \| A/G \| 89 (42.8%) \| 67 (32.2%) \| **0.61 (0.41-0.93)** \| \| G/G \| 18 (8.7%) \| 12 (5.8%) \| 0.56 (0.25-1.22) \| \| Dominant \| A/A \| 101 (48.6%) \| 129 (62%) \| 1.00 \| 0.012 \| 571.3 \| 587.4 \| \| A/G-G/G \| 107 (51.4%) \| 79 (38%) \| **0.60 (0.41-0.90)** \| \| Recessive \| A/A-A/G \| 190 (91.3%) \| 196 (94.2%) \| 1.00 \| 0.32 \| 576.6 \| 592.8 \| \| G/G \| 18 (8.7%) \| 12 (5.8%) \| 0.68 (0.32-1.46) \| \| Overdominant \| A/A-G/G \| 119 (57.2%) \| 141 (67.8%) \| 1.00 \| 0.041 \| 573.4 \| 589.6 \| \| A/G \| 89 (42.8%) \| 67 (32.2%) \| **0.66 (0.44-0.98)** \| \| Log-additive \| --- \| --- \| --- \| **0.68 (0.50-0.93)** \| 0.016 \| 571.8 \| 587.9 \|  \| **Interaction analysis with covariate sex** \| \| --- \|  \| **rs2494752 and sex cross-classification interaction table (n=416, adjusted by age)** \| \| \| \| \| \| \| \| --- \| --- \| --- \| --- \| --- \| --- \| --- \| \|  \| **female** \| \| \| **male** \| \| \| \|  \| **status=Ca** \| **status=Co** \| **OR (95% CI)** \| **status=Ca** \| **status=Co** \| **OR (95% CI)** \| \| **A/A** \| 61 \| 85 \| 1.00 \| 40 \| 44 \| 0.77 (0.45-1.33) \| \| **A/G** \| 53 \| 40 \| **0.57 (0.33-0.96)** \| 36 \| 27 \| **0.54 (0.30-0.98)** \| \| **G/G** \| 13 \| 5 \| **0.29 (0.10-0.86)** \| 5 \| 7 \| 1.08 (0.32-3.62) \| \| **Interaction p-value:**0.16 \| \| \| \| \| \| \|  \| **sex within rs2494752 (n=416, adjusted by age)** \| \| \| --- \| --- \| \| **A/A** \| \|  \| **status=Ca** \| **status=Co** \| **OR (95% CI)** \| \| --- \| --- \| --- \| --- \| \| **female** \| 61 \| 85 \| 1.00 \| \| **male** \| 40 \| 44 \| 0.77 (0.45-1.33) \| \| \| **A/G** \| \|  \| **status=Ca** \| **status=Co** \| **OR (95% CI)** \| \| --- \| --- \| --- \| --- \| \| **female** \| 53 \| 40 \| 1.00 \| \| **male** \| 36 \| 27 \| 0.95 (0.50-1.82) \| \| \| **G/G** \| \|  \| **status=Ca** \| **status=Co** \| **OR (95% CI)** \| \| --- \| --- \| --- \| --- \| \| **female** \| 13 \| 5 \| 1.00 \| \| **male** \| 5 \| 7 \| 3.73 (0.79-17.68) \| \| \| **Test for interaction in the trend:**0.11 \| \|  \| **rs2494752 within sex (n=416, adjusted by age)** \| \| \| --- \| --- \| \| **female** \| \|  \| **status=Ca** \| **status=Co** \| **OR (95% CI)** \| \| --- \| --- \| --- \| --- \| \| **A/A** \| 61 \| 85 \| 1.00 \| \| **A/G** \| 53 \| 40 \| **0.57 (0.33-0.96)** \| \| **G/G** \| 13 \| 5 \| **0.29 (0.10-0.86)** \| \| \| **male** \| \|  \| **status=Ca** \| **status=Co** \| **OR (95% CI)** \| \| --- \| --- \| --- \| --- \| \| **A/A** \| 40 \| 44 \| 1.00 \| \| **A/G** \| 36 \| 27 \| 0.70 (0.36-1.36) \| \| **G/G** \| 5 \| 7 \| 1.40 (0.41-4.86) \| \| \| **Test for interaction in the trend:**0.16 \| \| |  |
| **SNP:**rs5811155 |  |
| **Percentage of typed samples:**416/420 (99.05%)   \| **rs5811155 allele frequencies (n=416)** \| \| \| \| \| \| \| \| --- \| --- \| --- \| --- \| --- \| --- \| --- \| \|  \| **All subjects** \| \| **status=Ca** \| \| **status=Co** \| \| \| **Allele** \| **Count** \| **Proportion** \| **Count** \| **Proportion** \| **Count** \| **Proportion** \| \| C \| 606 \| 0.73 \| 284 \| 0.68 \| 322 \| 0.77 \| \| T \| 226 \| 0.27 \| 132 \| 0.32 \| 94 \| 0.23 \|  \| **rs5811155 genotype frequencies (n=420)** \| \| \| \| \| \| \| \| --- \| --- \| --- \| --- \| --- \| --- \| --- \| \|  \| **All subjects** \| \| **status=Ca** \| \| **status=Co** \| \| \| **Genotype** \| **Count** \| **Proportion** \| **Count** \| **Proportion** \| **Count** \| **Proportion** \| \| C/C \| 222 \| 0.53 \| 96 \| 0.46 \| 126 \| 0.61 \| \| C/T \| 162 \| 0.39 \| 92 \| 0.44 \| 70 \| 0.34 \| \| T/T \| 32 \| 0.08 \| 20 \| 0.1 \| 12 \| 0.06 \| \| NA \| 4 \| --- \| 0 \| --- \| 0 \| --- \|  \| **rs5811155 exact test for Hardy-Weinberg equilibrium (n=416)** \| \| \| \| \| \| \| \| --- \| --- \| --- \| --- \| --- \| --- \| --- \| \|  \| **N11** \| **N12** \| **N22** \| **N1** \| **N2** \| **P-value** \| \| **All subjects** \| 222 \| 162 \| 32 \| 606 \| 226 \| 0.71 \| \| **status=Ca** \| 96 \| 92 \| 20 \| 284 \| 132 \| 0.87 \| \| **status=Co** \| 126 \| 70 \| 12 \| 322 \| 94 \| 0.56 \|  \| **rs5811155 association with response status (n=416, adjusted by sex+age)** \| \| \| \| \| \| \| \| \| --- \| --- \| --- \| --- \| --- \| --- \| --- \| --- \| \| **Model** \| **Genotype** \| **status=Ca** \| **status=Co** \| **OR (95% CI)** \| **P-value** \| **AIC** \| **BIC** \| \| Codominant \| C/C \| 96 (46.1%) \| 126 (60.6%) \| 1.00 \| 0.027 \| 572.4 \| 592.5 \| \| C/T \| 92 (44.2%) \| 70 (33.6%) \| **0.61 (0.40-0.92)** \| \| T/T \| 20 (9.6%) \| 12 (5.8%) \| 0.50 (0.23-1.07) \| \| Dominant \| C/C \| 96 (46.1%) \| 126 (60.6%) \| 1.00 \| 0.0082 \| 570.7 \| 586.8 \| \| C/T-T/T \| 112 (53.9%) \| 82 (39.4%) \| **0.59 (0.40-0.87)** \| \| Recessive \| C/C-C/T \| 188 (90.4%) \| 196 (94.2%) \| 1.00 \| 0.2 \| 576 \| 592.1 \| \| T/T \| 20 (9.6%) \| 12 (5.8%) \| 0.62 (0.29-1.31) \| \| Overdominant \| C/C-T/T \| 116 (55.8%) \| 138 (66.3%) \| 1.00 \| 0.046 \| 573.7 \| 589.8 \| \| C/T \| 92 (44.2%) \| 70 (33.6%) \| **0.67 (0.45-0.99)** \| \| Log-additive \| --- \| --- \| --- \| **0.66 (0.48-0.90)** \| 0.0086 \| 570.7 \| 586.9 \|  \| **Interaction analysis with covariate sex** \| \| --- \|  \| **rs5811155 and sex cross-classification interaction table (n=416, adjusted by age)** \| \| \| \| \| \| \| \| --- \| --- \| --- \| --- \| --- \| --- \| --- \| \|  \| **female** \| \| \| **male** \| \| \| \|  \| **status=Ca** \| **status=Co** \| **OR (95% CI)** \| **status=Ca** \| **status=Co** \| **OR (95% CI)** \| \| **C/C** \| 57 \| 84 \| 1.00 \| 39 \| 42 \| 0.71 (0.41-1.24) \| \| **C/T** \| 57 \| 41 \| **0.52 (0.30-0.88)** \| 35 \| 29 \| 0.57 (0.31-1.03) \| \| **T/T** \| 13 \| 5 \| **0.28 (0.09-0.82)** \| 7 \| 7 \| 0.75 (0.25-2.29) \| \| **Interaction p-value:**0.2 \| \| \| \| \| \| \|  \| **sex within rs5811155 (n=416, adjusted by age)** \| \| \| --- \| --- \| \| **C/C** \| \|  \| **status=Ca** \| **status=Co** \| **OR (95% CI)** \| \| --- \| --- \| --- \| --- \| \| **female** \| 57 \| 84 \| 1.00 \| \| **male** \| 39 \| 42 \| 0.71 (0.41-1.24) \| \| \| **C/T** \| \|  \| **status=Ca** \| **status=Co** \| **OR (95% CI)** \| \| --- \| --- \| --- \| --- \| \| **female** \| 57 \| 41 \| 1.00 \| \| **male** \| 35 \| 29 \| 1.10 (0.58-2.08) \| \| \| **T/T** \| \|  \| **status=Ca** \| **status=Co** \| **OR (95% CI)** \| \| --- \| --- \| --- \| --- \| \| **female** \| 13 \| 5 \| 1.00 \| \| **male** \| 7 \| 7 \| 2.73 (0.62-12.00) \| \| \| **Test for interaction in the trend:**0.078 \| \|  \| **rs5811155 within sex (n=416, adjusted by age)** \| \| \| --- \| --- \| \| **female** \| \|  \| **status=Ca** \| **status=Co** \| **OR (95% CI)** \| \| --- \| --- \| --- \| --- \| \| **C/C** \| 57 \| 84 \| 1.00 \| \| **C/T** \| 57 \| 41 \| **0.52 (0.30-0.88)** \| \| **T/T** \| 13 \| 5 \| **0.28 (0.09-0.82)** \| \| \| **male** \| \|  \| **status=Ca** \| **status=Co** \| **OR (95% CI)** \| \| --- \| --- \| --- \| --- \| \| **C/C** \| 39 \| 42 \| 1.00 \| \| **C/T** \| 35 \| 29 \| 0.79 (0.41-1.54) \| \| **T/T** \| 7 \| 7 \| 1.05 (0.33-3.33) \| \| \| **Test for interaction in the trend:**0.2 \| \| |  |
| **Multiple-SNP analysis** |  |
| \| **Linkage disequilibrium analysis** \| \| --- \|   **D statistic**   \|  \| rs2498786 \| rs2494752 \| rs5811155 \| \| --- \| --- \| --- \| --- \| \| rs2498786 \| . \| 0.1288 \| 0.1383 \| \| rs2494752 \| . \| . \| 0.189 \| \| rs5811155 \| . \| . \| . \|   **D' statistic**   \|  \| rs2498786 \| rs2494752 \| rs5811155 \| \| --- \| --- \| --- \| --- \| \| rs2498786 \| . \| 0.9157 \| 0.9997 \| \| rs2494752 \| . \| . \| 0.9996 \| \| rs5811155 \| . \| . \| . \|   **r statistic**   \|  \| rs2498786 \| rs2494752 \| rs5811155 \| \| --- \| --- \| --- \| --- \| \| rs2498786 \| . \| 0.7487 \| 0.7926 \| \| rs2494752 \| . \| . \| 0.9693 \| \| rs5811155 \| . \| . \| . \|   **P-values**   \|  \| rs2498786 \| rs2494752 \| rs5811155 \| \| --- \| --- \| --- \| --- \| \| rs2498786 \| . \| 0 \| 0 \| \| rs2494752 \| . \| . \| 0 \| \| rs5811155 \| . \| . \| . \| |  |
| \| **Haplotype analysis** \| \| --- \| |  |
| \| **Haplotype frequencies estimation (n=416)** \| \| \| \| \| \| \| \| \| --- \| --- \| --- \| --- \| --- \| --- \| --- \| --- \| \|  \| **rs2498786** \| **rs2494752** \| **rs5811155** \| **Total** \| **group.Ca** \| **group.Co** \| **Cumulative frequency** \| \| 1 \| C \| A \| C \| 0.7284 \| 0.6827 \| 0.774 \| 0.7284 \| \| 2 \| G \| G \| T \| 0.1779 \| 0.2163 \| 0.1394 \| 0.9063 \| \| 3 \| C \| G \| T \| 0.0817 \| 0.0841 \| 0.0793 \| 0.988 \| \| 4 \| G \| A \| T \| 0.012 \| 0.0168 \| 0.0072 \| 1 \| \| 5 \| C \| A \| T \| 0 \| 0 \| NA \| 1 \| |  |
| \| **Haplotype association with response (n=416, adjusted by sex+age)** \| \| \| \| \| \| \| \| \| \| --- \| --- \| --- \| --- \| --- \| --- \| --- \| --- \| --- \| \|  \| **rs2498786** \| **rs2494752** \| **rs5811155** \| **Freq** \| **OR (95% CI)** \| **P-value** \|  \|  \| \| 1 \| C \| A \| C \| 0.7284 \| 1.00 \| --- \|  \|  \| \| 2 \| G \| G \| T \| 0.1779 \| **0.56 (0.38 - 0.82)** \| 0.0035 \|  \|  \| \| 3 \| C \| G \| T \| 0.0817 \| 0.89 (0.55 - 1.42) \| 0.61 \|  \|  \| \| 4 \| G \| A \| T \| 0.012 \| 0.42 (0.10 - 1.70) \| 0.23 \|  \|  \| \| **Global haplotype association p-value: 0.02** \| \| \| \| \| \| \| \| \| |  |
| \| **Haplotype interaction analysis with covariate sex** \| \| --- \| |  |
| \| **Haplotype and sex cross-classification interaction table (n=416, adjusted by age)** \| \| \| \| \| --- \| --- \| --- \| --- \| \|  \|  \| **female** \| **male** \| \| **Haplotype** \| **Frequency** \| **OR (95% CI)** \| **OR (95% CI)** \| \| **CAC** \| 0.7284 \| 1.00 \| 0.67 (0.39 - 1.15) \| \| **CGT** \| 0.0817 \| 0.77 (0.42 - 1.40) \| 0.74 (0.33 - 1.65) \| \| **GAT** \| 0.012 \| 0.20 (0.02 - 1.84) \| 0.54 (0.08 - 3.46) \| \| **GGT** \| 0.1779 \| **0.41 (0.24 - 0.69)** \| 0.57 (0.32 - 1.02) \| \| **Interaction p-value:**0.24 \| \| \| \| |  |
| \| **Haplotypes within sex (n=416, adjusted by age)** \| \| \| \| \| --- \| --- \| --- \| --- \| \|  \|  \| **female** \| **male** \| \| **Haplotype** \| **Frequency** \| **OR (95% CI)** \| **OR (95% CI)** \| \| **CAC** \| 0.7284 \| 1.00 \| 1.00 \| \| **CGT** \| 0.0817 \| 0.77 (0.42 - 1.40) \| 1.10 (0.51 - 2.37) \| \| **GAT** \| 0.012 \| 0.20 (0.02 - 1.84) \| 0.80 (0.13 - 5.11) \| \| **GGT** \| 0.1779 \| **0.41 (0.24 - 0.69)** \| 0.85 (0.47 - 1.53) \| |  |
| \| **sex whithin haplotypes (n=416, adjusted by age)** \| \| \| \| \| --- \| --- \| --- \| --- \| \|  \|  \| **female** \| **male** \| \| **Haplotype** \| **Frequency** \| **OR (95% CI)** \| **OR (95% CI)** \| \| **CAC** \| 0.7284 \| 1.00 \| 0.67 (0.39 - 1.15) \| \| **CGT** \| 0.0817 \| 1.00 \| 0.96 (0.38 - 2.42) \| \| **GAT** \| 0.012 \| 1.00 \| 2.71 (0.16 - 47.09) \| \| **GGT** \| 0.1779 \| 1.00 \| 1.39 (0.72 - 2.67) \| |  |
