# Supplementary material for: Association between the AKT1 single nucleotide polymorphism (rs2498786, rs2494752 and rs5811155) and microscopic polyangiitis risk in a Chinese population
Source: Mol Genet Genomics. 2023 Apr 7;298(3):767–76. doi: 10.1007/s00438-023-02012-6 (PMC10133348; doi:10.1007/s00438-023-02012-6)
Supplement: Supplementary file 2 — Supplementary file2 (DOCX 13 KB) [file 438_2023_2012_MOESM2_ESM.docx]

**Supplement Table 1:** Information of AKT1 SNPs

| Loci | Position ( AKT1） | Functional Consequence | Vriant Type | Ref | Alt |
| --- | --- | --- | --- | --- | --- |
| rs2498786 | 2KB Upstream | upstream_transcript_variant | SNV | C | G |
| rs2494752 | 2KB Upstream | Upstream transcript variant | SNV | A | G |
| rs5811155 | 2KB Upstream | upstream_transcript_variant | Insertion |  | insT |
| rs2498801 | 500B Downstream | Non Coding Transcript Variant | SNV | T | C |
| rs2494732 | Intron | Intron Variant | SNV | T | C |
| rs1130233 | Exon | Synonymous Variant | SNV | C | T |
| rs2494737 | Intron | Intron Variant | SNV | T | A |
| rs1130214 | Intron | Intron Variant | SNV | C | A |

Note: Data was from SNP database of NCBI.
